# Iminoiodane- and Brønsted Base-Mediated Cross Dehydrogenative Coupling of Cyclic Ethers with 1,3-Dicarbonyl Compounds

**DOI:** 10.3390/molecules200713336

**Published:** 2015-07-22

**Authors:** Ciputra Tejo, Xiao Rong Sim, Bo Ra Lee, Benjamin James Ayers, Chung-Hang Leung, Dik-Lung Ma, Philip Wai Hong Chan

**Affiliations:** 1 Division of Chemistry and Biological Chemistry, School of Physical and Mathematical Sciences, Nanyang Technological University, Singapore 637371, Singapore; E-Mails: cipu0002@e.ntu.edu.sg (C.T.); xrsim1@e.ntu.edu.sg (X.R.S.); benayers@ntu.edu.sg (B.J.A.); 2 School of Chemistry, Monash University, Clayton, Victoria 3800, Australia; E-Mail: bora.lee@monash.edu; 3 State Key Laboratory of Quality Research in Chinese Medicine, Institute of Chinese Medical Sciences, University of Macau, Macao, China; E-Mail: duncanleung@umac.mo; 4 Department of Chemistry, Hong Kong Baptist University, Kowloon Tong, Hong Kong, China; E-Mail: edmondma@hkbu.edu.hk; 5 Department of Chemistry, University of Warwick, Coventry CV4 7AL, UK

**Keywords:** C–C bond formation, cross dehydrogenation coupling, 1,3-dicarbonyl compounds, iminoiodanes, metal-free catalysis

## Abstract

A one-pot, two-step approach to prepare 2-tetrahydrofuran and -pyran substituted 1,3-dicarbonyl compounds by PhI=NTs-mediated amination/Brønsted base-catalyzed cross dehydrogenative coupling (CDC) reaction of the cyclic ether and 1,3-dicarbonyl derivative under mild conditions is reported. The reaction is compatible with a variety of cyclic ethers and 1,3-dicarbonyl compounds, affording the corresponding coupled products in moderate to good yields of up to 80% over two steps.

## 1. Introduction

**Scheme 1:**
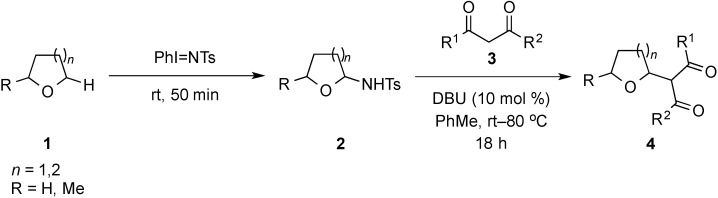
Iminoiodane-mediated CDC reaction of cyclic ethers with 1,3-dicarbonyl compounds.

Recently, there has been an increasing amount of attention toward the ultimate goal of the establishment of more sustainable organic transformations, owing to increased concerns over the impact of present chemical methods and processes on the living environment [1,2,3,4]. In this regard, the direct activation of carbon–hydrogen bonds in carbon–carbon bond forming CDC reactions has emerged as one of the most powerful and atom-economical methods in modern organic chemistry [5,6,7,8,9]. A number of transition metal salts, mainly those of Pd, Rh, Ru and Cu, in the presence of an oxidant, to effect these transformations at a variety of C–H bonds such as those at the benzylic, aryl, and alkyl C(sp^3^)–H positions have often been targeted [10,11,12,13,14,15,16,17,18,19,20,21,22,23,24,25,26,27,28,29,30,31,32,33,34,35]. In the case of the latter, this has included CDC reactions at the α-C–H bond of the heteroatom in ethers, amines, and sulfides with nucleophiles catalyzed by Fe or Cu salts [36,37,38,39,40,41,42,43,44,45,46,47,48,49,50,51,52,53,54,55,56,57,58,59,60,61]. More recently, the development of these reactions mediated by non-metal based catalysts has come under increasing scrutiny [62,63,64,65,66,67,68,69,70,71]. In the presence of an oxidant such as a peroxide, DDQ, TEMPO, dioxygen or hypervalent iodide reagent, a variety of carbon nucleophiles were shown to functionalize the α-carbon position of the heteroatom in amines and ethers [65,66,67,68,69,70,71,72,73,74,75,76,77,78,79,80,81]. As part of our interest in the chemistry of iminoiodanes, we wondered whether this class of I(III) compounds could mediate the α-functionalization of cyclic ethers by a carbon nucleophile under basic conditions. In doing so, we discovered THF, 2-methyl tetrahydrofuran and THP shown in Scheme 1 to undergo α-C–H bond amination by PhI=NTs [82,83,84,85,86,87,88,89,90,91,92,93,94,95,96,97,98,99,100,101,102,103,104,105,106,107,108,109]. This was followed by substitution at the aminal carbon center by 1,3-dicarbonyl compounds under the basic conditions. Herein, we report the details, this chemistry that provides access to 2-tetrahydrofuran and -pyran substituted 1,3-dicarbonyl compounds in up to 80% yield over two steps.

## 2. Results and Discussion

Our investigations began with the *in situ* generation of 2-tosylaminotetrahydrofuran **2a** from THF **1a** and PhI=NTs, which was obtained in 90% yield based on ^1^H-NMR measurements [95]. Subsequent treatment of this adduct with 3 equiv of ethyl benzoylacetate **3a** and 10 mol % of DBU as the catalyst in THF at room temperature for 18 h gave ethyl 3-oxo-3-phenyl-2-(tetrahydrofuran-2-yl)propanoate **4a** in 41% yield (Table 1, entry 1) [103,110,111,112]. Changing the solvent from THF to diethyl ether in the second step gave a comparable product yield (Table 1, entry 2). Our subsequent studies found that the use of dichloromethane and toluene in place of THF led to higher product yields of 79% and 78%, respectively (Table 1, entries 3 and 4). However, other bases, such as Et_3_N, DABCO, and MTBD, in place of DBU as the catalyst, afforded lower product yields of 61% or no reaction (Table 1, entries 5–7). With DBU as the base and toluene as the solvent, decreasing the amount of **3a** from 3 to 2 or 1 equiv led to comparable product yields of 80% and 73%, respectively (Table 1, entries 8 and 9). On the other hand, a lower product yield of 67% was observed on lowering the catalyst loading of DBU from 10 to 5 mol % (Table 1, entry 10). From these results, the one-pot reaction of **1a** and PhI=NTs at room temperature for 50 min followed by treating with 2 equiv of **3a** in the presence of 10 mol % of DBU catalyst in toluene at room temperature for 18 h was deemed to provide the optimal reaction conditions.

**Table 1:** Optimization of reaction conditions ^a^. 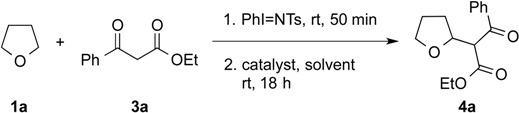

Entry	Catalyst (mol %)	Solvent	Yield (%) ^b^
1	DBU (10)	THF	41
2	DBU (10)	Et_2_O	54 ^c^
3	DBU (10)	CH_2_Cl_2_	79
4	DBU (10)	PhMe	78
5	Et_3_N (10)	PhMe	- ^d^
6	DABCO (10)	PhMe	- ^d^
7	MTBD (10)	PhMe	61
8 ^e^	**DBU (10)**	**PhMe**	**80**
9 ^f^	DBU (10)	PhMe	73 ^c^
10 ^e^	DBU (5)	PhMe	67

^a^ All reactions were carried out under N_2_(g) with 0.25 M of PhI=NTs in THF for 50 min followed by treatment with the appropriate reaction condition. ^b^ Isolated yield over two steps. ^c^ Yield was determined by ^1^H NMR analysis of crude mixture. ^d^ No reaction observed based on TLC and ^1^H NMR analysis of the crude mixture. ^e^ Two equiv of **3a** was used. ^f^ One equiv of **3a** was used.

**Figure 1:**
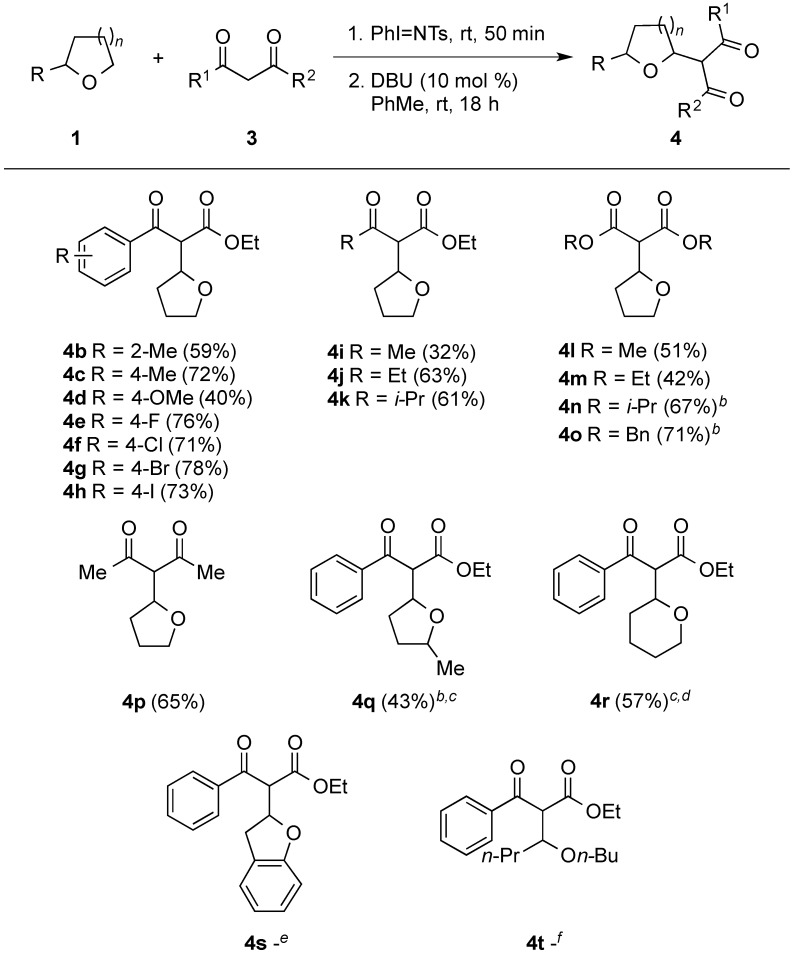
Iminoiodane-mediated CDC reactions of cyclic ethers **1** and 1,3-dicarbonyl compounds **3** ^a^.

^a^ All reactions were carried out under N_2_(g) with 0.25 M of PhI=NTs in ether for 50 min followed by treatment with 2 equiv of **3** and of 10 mol % DBU in toluene for 18 h. Isolated yields over two steps are in parentheses. ^b^ Reaction temperature = 40 °C. ^c^ Isolated yield is calculated based on the conversion of step one. Refer to experimental section for details. ^d^ Reaction temperature = 80 °C. ^e^ No reaction observed based on TLC and ^1^H-NMR analysis of the crude mixture. ^f^ Decomposition of starting material based on TLC and ^1^H-NMR analysis of the crude mixture.

To define the generality of the present procedure, a series of cyclic ethers **1** and 1,3-dicarbonyl compounds **3** were tested and the results are summarized in Figure 1. These experiments revealed that reaction of **1a** with a range of aryl-substituted β-ketoesters bearing electron-donating (**3b**–**d**) and electron-withdrawing (**3e**–**h**) groups proceeded well to afford the corresponding adducts **4b**–**h** in good yields of 40%–78%. Likewise, aliphatic-substituted β-ketoesters (**3i**–**k**) were well tolerated, furnishing the corresponding targets **4i**–**k** in yields of 32%–63%. The present methodology was also applicable to dialkyl malonates (**3l**–**o**), as well as the 1,3-dimethyl dione **3p** with the corresponding products **4l**–**p** provided in good yields of 42%–71%. This is notable, as existing transition metal-catalyzed CDC reactions of these types of 1,3-dicarbonyl compounds have been previously reported to be incompatible [60].

The influence of the cyclic ether coupling partner on the efficiency of the reaction was then assessed. For 2-methyltetrahydrofuran **1b** and THP **1c**, the reaction of these cyclic ethers with **3a** gave the corresponding adducts **4q** and **4r** in 43% and 57% yield, respectively. However, no reaction was observed when either 2,3-dihydrobenzofuran **1s** or dibutyl ether **1t** was treated with **3a**, under the standard conditions, with PhI=NTs and DBU. In the case of **1t**, decomposition of the α-aminated ether intermediate was observed by both TLC and ^1^H-NMR analysis of the crude reaction mixture.

**Scheme 2:**
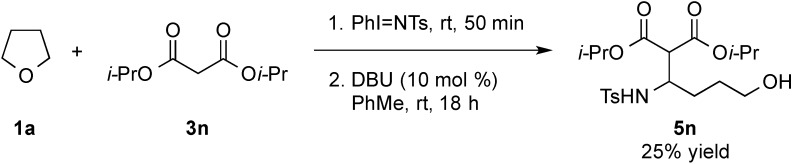
Reaction of **3n** under optimum conditions at room temperature.

**Figure 2:**
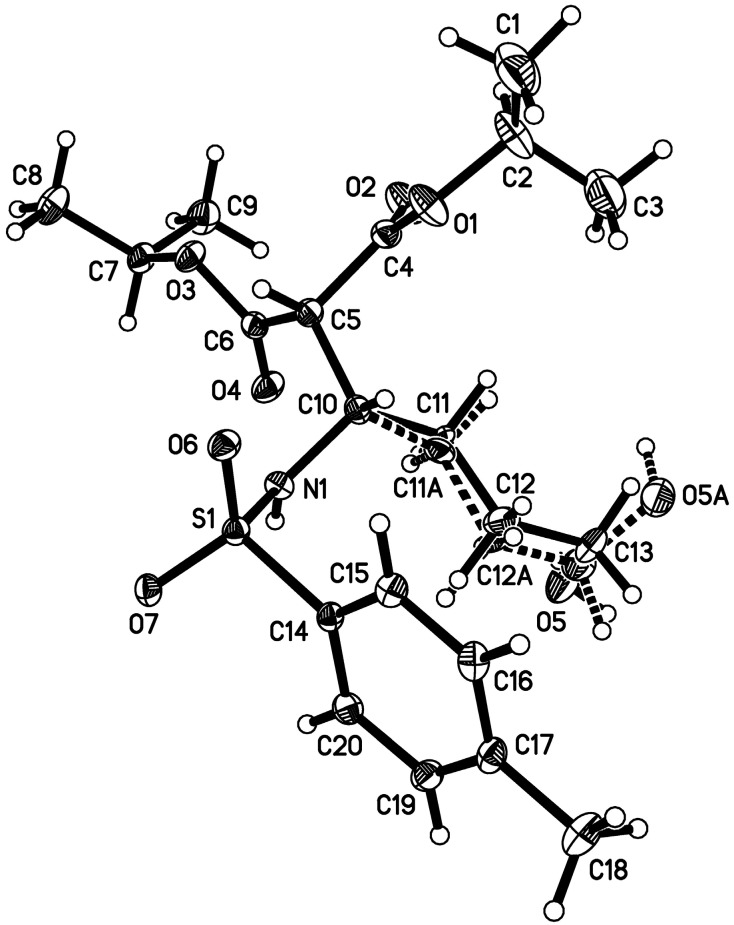
ORTEP drawing for **5n** with thermal ellipsoids at 50% probability level [119].

**Scheme 3:**
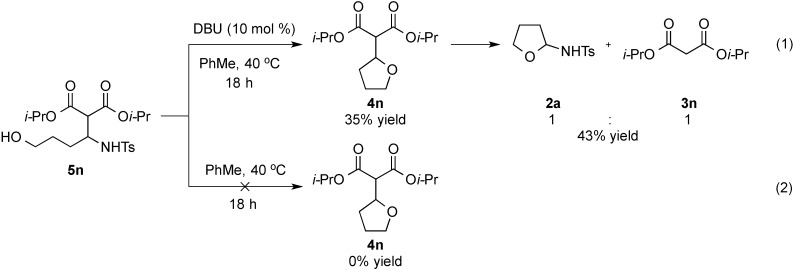
Control experiments with **5n** in the absence and presence of DBU.

At room temperature, reaction of **1a** with diisopropyl malonate **3n** was found to lead to **5n** being isolated in 25% yield (Scheme 2). The structure of compound **5n** was confirmed by single crystal X-ray analysis (Figure 2). The isolation of this acyclic adduct led us to speculate its possible involvement as an intermediate in the α-functionalization reaction. This was further supported by re-subjecting **5n** to 10 mol % of DBU under the standard conditions at 40 °C (Scheme 3, eq. 1). This test gave **4n** along with a 1:1 mixture of **2a** and **3n** in 35% and 43% yield, respectively, with the latter two adducts being obtained, presumably, from a competitive retro-Mannich-type pathway [113,114,115,116,117,118]. The role of DBU in mediating the cyclization of the 1,4 amino aldol was also supported by our findings showing the recovery of the substrate on treating it to the standard conditions in the absence of the Schiff base (Scheme 3, Equation (2)).

**Scheme 4:**
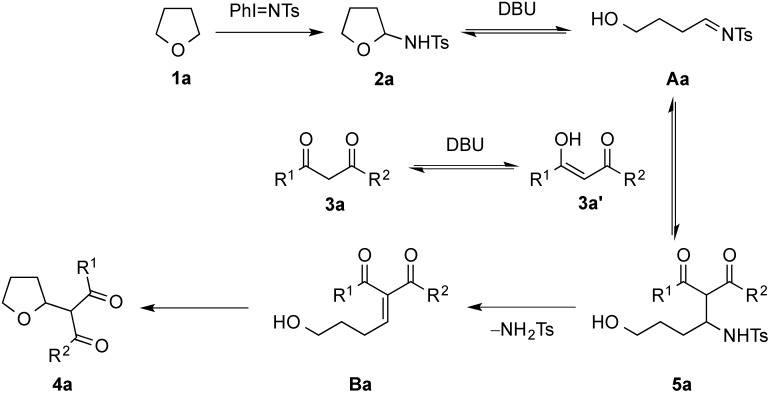
Proposed mechanism of CDC of cyclic ethers **1a** and 1,3-dicarbonyl compounds **3a**.

A tentative mechanism for the present iminoidane-mediated transformation under basic conditions is illustrated in Scheme 4. Using the reaction **1a** with **3a** as a representative example, this could involve formation of **2a** on treating the cyclic ether with the PhI=NTs [95,96]. While the possible amination pathway of this step remains presently unclear, the basic conditions provided by DBU may promote ring-opening of the adduct to give the 1,4-imino alcohol intermediate **Aa**. Nucleophilic attack at the imino carbon center of this substrate by the enolate of **3a** would deliver the amino alcohol **5a**. On base-mediated deamination, the ensuing 3-methylene β-keto ester **Ba** might undergo 5-exo-trig cyclization involving addition of the hydroxyl moiety to the alkene bond in the adduct to provide the product **4a**.

## 3. Experimental Section

### General Information

All reactions were performed in oven-dried glassware, under a N_2_(g) atmosphere at ambient temperatures, unless otherwise stated. Unless specified, all reagents and starting materials were purchased from commercial sources and used as received. PhI=NTs was prepared following literature procedures [120]. Toluene and THF were distilled over sodium/benzophenone, and 2-methyltetrahydrofuran, tetrahydropyran, CH_2_Cl_2_ and MeCN were purified prior to use by distilling over CaH_2_. Analytical thin layer chromatography (TLC) was performed using Merck 60 F254 pre-coated silica gel plates (Merck, Darmstadt, Germany). Visualization was achieved by UV-Vis light (254 nm) followed by treatment with ninhydrin stain and heating. Flash chromatography was performed using Merck silica gel 60 and a gradient solvent system (EtOAc/*n*-hexane as eluent). Unless otherwise stated, ^1^H- and ^13^C-NMR spectra were measured on a Bruker AV300 or AV400 NMR spectrometer (Bruker, Fällanden, Switzerland), and chemical shifts (ppm) were recorded in CDCl_3_ solution with tetramethylsilane (TMS) as the internal reference standard. ^1^H-NMR product yields were determined with CH_2_Br_2_ as the internal reference standard. Multiplicities are given as s (singlet), d (doublet), t (triplet), q (quartet), dt (doublet of triplets), or m (multiplet). The number of protons (*n*) for a given resonance is indicated by *n*H, and coupling constants are reported as a *J* value in Hz. Infrared spectra were recorded on a Shimadzu IR Prestige-21 FTIR spectrometer (Shimadzu, Kyoto, Japan). Solid samples were examined as a thin film between NaCl salt plates. Low resolution mass spectra were determined on a LCQ XP MAX mass spectrometer (ThermoFisher Scientific, San Jose, CA, USA) and reported as a ratio of mass to charge (*m*/*z*). High resolution mass spectra (HRMS) were obtained using a Finnigan MAT95XP LC/HRMS Q-TOF mass spectrometer (Waters, Manchester, UK). The ^1^H- and ^13^C-NMR spectra of products **4a**–**r** and compound **5n** is available in the Supplementary Materials.

Representative procedure for CDC of tetrahydronfuran **1a** with 1,3-dicarbonyl compounds **3a**: Tetrahydrofuran **1a** (1 mL) was added to PhI=NTs (93 mg, 0.25 mmol) in a 5 mL round-bottomed flask and stirred for 50 min at room temperature. The solvent was then removed under reduced pressure and the flask back filled with N_2_(g). To the crude mixture, 1,3-dicarbonyl compound **3a** (0.50 mmol, 2 equiv), DBU (4 μL, 0.025 mmol), and PhMe (1 mL) was subsequently added. The reaction was stirred for 18 h at room temperature or 40 °C. Upon completion of the reaction, as judged by TLC analysis, the crude mixture was purified by flash column chromatography (eluent: *n*-hexane/EtOAc, 5:1–4:1) to give the corresponding product **4a**.

*Ethyl 3-oxo-3-phenyl-2-(tetrahydrofuran-2-yl)propanoate* (**4a**) [60]. Wt 52.2 mg; yield 80%; obtained as two diastereomers with ratio of 1.3:1; yellow oil; ^1^H-NMR (400 MHz, CDCl_3_) δ 1.15–1.20 (m, 6H), 1.47–1.56 (m, 1H), 1.74–1.81 (m, 1H), 1.83–1.98 (m, 4H), 2.16–2.27 (m, 2H), 3.70–3.91 (m, 4H), 4.11–4.20 (m, 4H), 4.41 (d, *J* = 8.8 Hz, 1H), 4.46 (d, *J* = 8.8 Hz, 1H), 4.65–4.73 (m, 2H), 7.45–7.50 (m, 2H), 7.56–7.61 (m, 1H), 8.02–8.05 (m, 2H); ^13^C-NMR (100 MHz, CDCl_3_) δ 13.9, 14.0, 25.4, 25.5, 29.7, 30.0, 30.2, 59.3, 60.2, 61.4, 61.6, 68.1, 68.2, 77.7, 78.1, 128.6, 128.7, 128.7, 133.4, 133.7, 136.4, 136.8, 167.5, 167.9, 193.3, 193.6.

*Ethyl 3-oxo-2-(tetrahydrofuran-2-yl)-3-(o-tolyl)propanoate* (**4b**). Wt 41.1 mg; yield 59%; obtained as two diastereomers with ratio of 1.2:1; yellow oil; ^1^H-NMR (300 MHz, CDCl_3_) δ 1.14 (t, *J* = 5.6 Hz, 3H), 1.93 (t, *J* = 5.6 Hz, 3H), 1.52–1.64 (m, 1H), 1.68–1.80 (m, 1H), 1.86–1.97 (m, 4H), 2.17–2.27 (m, 2H), 2.49 (s, 3H), 2.50 (s, 3H), 3.69–3.91 (m, 4H), 4.09–4.21 (m, 4H), 4.31 (d, *J* = 2.7 Hz, 1H), 4.34 (d, *J* = 3.0 Hz, 1H), 4.58–4.66 (m, 2H), 7.24–7.30 (m, 4H), 7.35–7.42 (m, 2H), 7.74–7.89 (m, 2H); ^13^C-NMR (75 MHz, CDCl_3_) δ 13.9, 14.0, 20.9, 21.1, 25.5, 30.1, 30.2, 61.3, 61.4, 61.8, 62.3, 68.0, 68.1, 78.1, 78.1, 125.5, 125.7, 128.7, 129.0, 131.5, 131.8, 131.9, 132.0, 138.8, 138.9, 167.6, 167.9, 196.7, 197.2; IR (NaCl, neat) ν 2979, 2930, 1740, 1688, 1456 cm^−1^; HRMS (ESI) calcd for C_16_H_20_NaO_4_ [M + Na]^+^ 299.1259, found 299.1268.

*Ethyl 3-oxo-2-(tetrahydrofuran-2-yl)-3-(p-tolyl)propanoate* (**4c**). Wt 49.9 mg; yield 72%; obtained as two diastereomers with ratio of 1.2:1; yellow oil; ^1^H-NMR (300 MHz, CDCl_3_) δ 1.15–1.20 (m, 6H), 1.44–1.56 (m, 1H), 1.71–1.81 (m, 1H), 1.83–1.98 (m, 4H), 2.13–2.27 (m, 2H), 2.40 (s, 3H), 2.41 (s, 3H), 3.68–3.91 (m, 4H), 4.10–4.20 (m, 4H), 4.38 (d, *J* = 9 Hz, 1H), 4.42 (d, *J* = 9 Hz, 1H), 4.62–4.73 (m, 2H), 7.25–7.28 (m, 4H), 7.91–7.95 (m, 4H); ^13^C-NMR (75 MHz, CDCl_3_) δ 14.0, 21.7, 25.4, 25.5, 30.0, 30.2, 59.2, 60.1, 61.4, 61.5, 68.1, 68.1, 77.7, 78.1, 128.9, 129.0, 129.3, 129.4, 134.0, 134.4, 144.4, 144.7, 167.7, 168.0, 192.8, 193.1; IR (NaCl, neat) ν 2980, 1732, 1682, 1607 cm^−1^; HRMS (ESI) calcd for C_16_H_21_O_4_ [M + H]^+^ 277.1440, found 277.1439.

*Ethyl 3-(4-methoxyphenyl)-3-oxo-2-(tetrahydrofuran-2-yl)propanoate* (**4d**). Wt 31.6 mg; yield 40%; obtained as two diastereomers with ratio of 1.4:1; yellow oil; ^1^H-NMR (400 MHz, CDCl_3_) δ 1.16–1.21 (m, 6H), 1.47–1.54 (m, 1H), 1.74–1.81 (m, 1H) 1.85–2.00 (m, 4H), 2.14–2.24 (m, 2H), 3.70–3.88 (m, 10H), 4.11–4.20 (m, 4H), 4.36 (d, *J* = 9.2 Hz, 1H), 4.40 (d, *J* = 9.2 Hz, 1H), 4.64–4.73 (m, 2H), 6.93–6.96 (m, 4H), 8.01–8.04 (m, 4H); ^13^C-NMR (100 MHz, CDCl_3_) δ 14.0, 14.0, 25.4, 25.5, 30.0, 30.2, 55.5, 55.5, 59.0, 60.0, 61.4, 61.5, 68.1, 68.2, 77.7, 78.2, 113.8, 113.9, 129.5, 129.8, 131.2, 131.3, 163.9, 164.1, 167.8, 168.2, 191.6, 191.9; IR (NaCl, neat) ν 2978, 2938, 1736, 1674, 1601, 1574, 1512 cm^−1^; HRMS (ESI) calcd for C_16_H_21_O_5_ [M + H]^+^ 293.1389, found 293.1400.

*Ethyl 3-(4-fluorophenyl)-3-oxo-2-(tetrahydrofuran-2-yl)propanoate* (**4e**). Wt 53.1 mg; yield 76%; obtained as two diastereomers with ratio of 1.1:1; yellow oil; ^1^H-NMR (300 MHz, CDCl_3_) δ 1.16–1.22 (m, 6H), 1.46–1.58 (m, 1H), 1.72–1.82 (m, 1H), 1.84–1.99 (m, 4H), 2.17–2.27 (m, 2H), 3.69–3.91 (m, 4H), 4.12–4.21 (m, 4H), 4.37 (d, *J* = 8.7 Hz, 1H), 4.39 (d, *J* = 9.0 Hz, 1H), 4.62–4.72 (m, 2H), 7.11–7.18 (m, 4H), 8.04–8.10 (m, 4H); ^13^C-NMR (75 MHz, CDCl_3_) δ 13.9, 25.4, 30.1, 30.2, 59.4, 60.2, 61.5, 61.7, 68.1, 68.2, 77.7, 78.0, 115.6, 115.8, 115.9, 116.0, 131.4, 131.5, 131.6, 131.6, 167.4, 167.8, 191.8, 192.1; IR (NaCl, neat) ν 2980, 1735, 1684, 1597 cm^−1^; HRMS (ESI) calcd for C_15_H_17_FNaO_4_ [M + Na]^+^ 303.1009, found 303.0999.

*Ethyl 3-(4-chlorophenyl)-3-oxo-2-(tetrahydrofuran-2-yl)propanoate* (**4f**). Wt 52.9 mg; yield 71%; obtained as two diastereomers with ratio of 1:1; yellow oil; ^1^H-NMR (300 MHz, CDCl_3_) δ 1.16–1.21 (m, 6H), 1.46–1.58 (m, 1H), 1.69–1.79 (m, 1H), 1.8–1.99 (m, 4H), 2.15–2.27 (m, 2H), 3.69–3.90 (m, 4H), 4.11–4.21 (m, 4H), 4.36 (d, *J* = 8.7 Hz, 1H), 4.38 (d, *J* = 9.3 Hz, 1H), 4.61–4.72 (m, 2H), 7.43–7.47 (m, 4H), 7.95–8.00 (m, 4H); ^13^C-NMR (75 MHz, CDCl_3_) δ 14.0, 25.4, 30.1, 30.2, 59.4, 60.2, 61.6, 61.7, 68.1, 68.2, 77.7, 77.9, 128.9, 129.1, 130.2, 130.2, 134.7, 135.2, 140.0, 140.3, 167.3, 167.7, 192.2, 192.5; IR (NaCl, neat) ν 2980, 1736, 1684, 1589 cm^−1^; HRMS (ESI) calcd for C_15_H_17_ClNaO_4_ [M + Na]^+^ 319.0713, found 319.0723.

*Ethyl 3-(4-bromophenyl)-3-oxo-2-(tetrahydrofuran-2-yl)propanoate* (**4g**). Wt 66.1 mg; yield 78%; obtained as two diastereomers with ratio of 1.2:1; yellow oil; ^1^H-NMR (300 MHz, CDCl_3_) δ 1.16–1.21 (m, 6H), 1.46–1.58 (m, 1H), 1.69–1.79 (m, 1H) 1.81–1.99 (m, 4H), 2.15–2.27 (m, 2H), 3.68–3.90 (m, 4H), 4.10–4.21 (m, 4H), 4.36 (d, *J* = 8.7 Hz, 1H) 4.37 (d, *J* = 9.3 Hz, 1H), 4.61–4.71 (m, 2H), 7.60–7.64 (m, 4H), 7.87–7.92 (m, 4H); ^13^C-NMR (75 MHz, CDCl_3_) δ 14.0, 25.4, 30.1, 30.2, 59.4, 60.1, 61.6, 61.7, 68.1, 68.2, 77.7, 77.9, 128.8, 129.1, 130.2, 130.3, 131.9, 132.1, 135.1, 135.6, 167.3, 167.7, 192.4, 192.8; IR (NaCl, neat) ν 2980, 1738, 1682 cm^−1^; HRMS (ESI) calcd for C_15_H_17_^79^BrNaO_4_ [M + Na]^+^ 363.0208, found 363.0222.

*Ethyl 3-(4-iodophenyl)-3-oxo-2-(tetrahydrofuran-2-yl)propanoate* (**4h**). Wt 70.4 mg; yield 73%; obtained as two diastereomers with ratio of 1.2:1; yellow oil; ^1^H-NMR (300 MHz, CDCl_3_) δ 1.16–1.21 (m, 6H), 1.45–1.57 (m, 1H), 1.69–1.80 (m, 1H), 1.84–1.98 (m, 4H), 2.14–2.27 (m, 2H), 3.68–3.90 (m, 4H), 4.05–4.25 (m, 4H), 4.34 (d, *J* = 8.7 Hz, 1H), 4.36 (d, *J* = 9.0 Hz, 1H), 4.60–4.71 (m, 2H), 7.71–7.75 (m, 4H), 7.82–7.86 (m, 4H); ^13^C-NMR (75 MHz, CDCl_3_) δ 14.0, 25.4, 30.1, 30.2, 59.4, 60.0, 61.6, 61.7, 68.1, 68.2, 77.7, 77.9, 101.7, 102.1, 130.1, 130.2, 135.6, 136.1, 138.0, 138.1, 167.3, 167.6, 192.7, 193.1; IR (NaCl, neat) ν 2978, 1732, 1682, 1582 cm^−1^; HRMS (ESI) calcd for C_15_H_17_INaO_4_[M + Na]^+^ 411.0069, found 411.0086.

*Ethyl 3-oxo-2-(tetrahydrofuran-2-yl)butanoate* (**4i**) [60]. Wt 16.0 mg; yield 32%; obtained as two diastereomers with ratio of 1.7:1; yellow oil; ^1^H-NMR (400 MHz, CDCl_3_) δ 1.25–1.31 (m, 6H), 1.55–1.69 (m, 2H), 1.88–1.95 (m, 4H), 2.11–2.22 (m, 2H), 2.25 (s, 3H), 2.31 (s, 3H), 3.51 (d, *J* = 9.2 Hz, 1H), 3.58 (d, *J* = 8.4 Hz, 1H), 3.72–3.86 (m, 4H), 4.16–4.26 (m, 4H), 4.41–4.48 (m, 2H); ^13^C-NMR (100 MHz, CDCl_3_) δ 14.0, 25.3, 25.5, 29.7, 29.8, 29.9, 30.4, 61.4, 61.5, 65.0, 65.4, 68.0, 68.2, 77.2, 167.5, 167.9, 201.5, 202.1.

*Ethyl 3-oxo-2-(tetrahydrofuran-2-yl)pentanoate* (**4j**). Wt 33.8 mg; yield 63%; obtained as two diastereomers with ratio of 1.7:1; yellow oil; ^1^H-NMR (300 MHz, CDCl_3_) δ 1.03–1.10 (m, 6H), 1.23–1.30 (m, 6H), 1.52–1.67 (m, 2H), 1.85–1.95 (m, 4H), 2.08–2.24 (m, 2H), 2.52–2.68 (m, 4H), 3.55 (d, *J* = 9.6 Hz, 1H), 3.61 (d, *J* = 8.7 Hz, 1H), 3.72–3.87 (m, 4H), 4.13–4.26 (m, 4H), 4.40–4.48 (m, 2H); ^13^C-NMR (75 MHz, CDCl_3_) δ 7.37, 7.46, 14.0, 25.3, 25.5, 29.8, 30.4, 36.2, 36.3, 61.3, 61.4, 64.0, 64.3, 68.0, 68.1, 77.2, 77.4, 167.6, 168.0, 204.1, 204.6.

*Ethyl 4-methyl-3-oxo-2-(tetrahydrofuran-2-yl)pentanoate* (**4k**). Wt 35.0 mg; yield 61%; diastereomer ratio could not be determined; ^1^H-NMR (300 MHz, CDCl_3_) δ 1.10–1.16 (m, 6H) 1.23–1.29 (m, 3H), 1.47–1.66 (m, 1H), 1.85–1.94 (m, 2H), 2.09–2.23 (m, 1H), 2.75–2.87 (m, 1H), 3.69–3.87 (m, 3H), 4.10–4.24 (m, 2H), 4.40–4.49 (m, 1H); ^13^C-NMR (75 MHz, CDCl_3_) δ 14.0, 14.0, 17.7, 17.8, 17.8, 18.2, 25.3, 25.5, 29.9, 30.3, 41.2, 41.7, 61.2, 61.4, 62.0, 62.5, 68.0, 68.0, 77.5, 77.8, 167.4, 167.7, 207.4, 207.7; IR (NaCl, neat) ν 2976, 2938, 1744, 1713, 1636 cm^−1^; HRMS (ESI) calcd for C_12_H_20_NaO_4_ [M + Na]^+^ 251.1259, found 251.1262.

*Dimethyl 2-(tetrahydrofuran-2-yl)malonate* (**4l**) [121]. Wt 25.7 mg; yield 51%; yellow oil; ^1^H-NMR (300 MHz, CDCl_3_) δ 1.66–1.78 (m, 1H), 1.88–1.97 (m, 2H), 2.11–2.22 (m, 1H), 3.49 (d, *J* = 9 Hz, 1H), 3.74 (s, 3H), 3.77 (s, 3H), 3.79–3.88 (m, 1H), 4.46 (dt, *J* = 8.7, 6.9 Hz, 1H); ^13^C-NMR (75 MHz, CDCl_3_) δ 25.4, 29.9, 52.5, 52.6, 57.1, 68.3, 77.0, 167.6, 170.0.

*Diethyl 2-(tetrahydrofuran-2-yl)malonate* (**4m**) [121]. Wt 24.2 mg; yield 42%; colourless oil; ^1^H-NMR (300 MHz, CDCl_3_) δ 1.24–1.30 (m, 6H), 1.68–1.79 (m, 1H), 1.88–1.97 (m, 2H), 2.11–2.22 (m, 1H), 3.44 (d, *J* = 9.3 Hz, 1H), 3.74–3.90 (m, 2H), 4.16–4.27 (m, 4H), 4.54 (dt, *J* = 9.0, 6.9 Hz, 1H); ^13^C-NMR (75 MHz, CDCl_3_) δ 14.0, 25.4, 29.9, 57.5, 61.4, 61.4, 68.2, 77.0, 167.2, 167.6.

*Diisopropyl 2-(tetrahydrofuran-2-yl)malonate* (**4n**). Wt 43.5 mg; yield 67%; colourless oil; ^1^H-NMR (300 MHz, CDCl_3_) δ 1.23–1.27 (m, 12H), 1.68–1.79 (m, 1H), 1.87–1.96 (m, 2H), 2.09–2.20 (m, 1H), 3.37 (d, *J* = 9.0 Hz, 1H), 3.74–3.89 (m, 2H), 4.39–4.47 (dt, *J* = 9.0, 6.9 Hz, 1H), 4.99–5.16 (m, 2H); ^13^C-NMR (75 MHz, CDCl_3_) δ 21.5, 21.6, 21.6, 25.4, 29.8, 57.8, 68.1, 68.8, 68.9, 76.9, 166.8, 167.1; IR (NaCl, neat) ν 2982, 2878, 1748, 1732, 1636 cm^−1^; HRMS (ESI) calcd for C_13_H_22_NaO_5_ [M + Na]^+^ 281.1365, found 281.1365.

*Dibenzyl 2-(tetrahydrofuran-2-yl)malonate* (**4o**). Wt 63.3 mg; yield 71%; colourless oil; ^1^H-NMR (400 MHz, CDCl_3_) δ 1.67–1.76 (m, 1H), 1.84–1.90 (m, 2H), 2.07–2.15 (m, 1H), 3.57 (d, *J* = 9.2 Hz, 1H), 3.72–3.85 (m, 2H), 4.50 (dt, *J* = 9.2, 6.8 Hz, 1H) 5.13 (s, 2H), 5.18 (s, 2H), 7.28–7.35 (m, 10H); ^13^C-NMR (100 MHz, CDCl_3_) δ 25.5, 29.9, 57.4, 67.1, 68.3, 77.0, 128.1, 128.2, 128.2, 128.4, 128.5, 128.6, 135.2, 135.5, 166.9, 167.3; IR (NaCl, neat) ν 3065, 3034, 2955, 2876, 1732, 1636 cm^−1^; HRMS (ESI) calcd for C_21_H_22_NaO_5_ [M + Na]^+^ 377.1365, found 377.1375.

*3-(Tetrahydrofuran-2-yl)pentane-2,4-dione* (**4p**) [121]. Wt 27.5 mg; yield 65%; yellow oil; ^1^H-NMR (400 MHz, CDCl_3_) δ 1.40–1.49 (m, 1H), 1.87–1.94 (m, 2H), 2.10–2.20 (m, 1H), 2.21 (s, 3H), 2.26 (s, 3H), 3.70–3.74 (m, 2H), 3.81–3.86 (m, 1H), 4.50 (m, *J* = 9.2, 6.8 Hz, 1H); ^13^C-NMR (100 MHz, CDCl_3_) δ 25.3, 29.5, 30.2, 30.4, 67.9, 74.3, 77.5, 202.2, 202.8.

*Ethyl 2-(5-methyltetrahydrofuran-2-yl)-3-oxo-3-phenylpropanoate* (**4q**). 2-Methyltetrahydrofuran **1b** (2 mL) was added to PhI=NTs (186 mg, 0.50 mmol) in a 5 mL round-bottomed flask and stirred for 50 min at room temperature (50% conversion based on ^1^H-NMR analysis). The solvent was removed under reduced pressure and the flask back filled with N_2_(g). To the crude mixture, ethyl benzoylacetate **3a** (87 μL, 0.50 mmol) and DBU (4 μL, 0.025 mmol) were subsequently added. In the absence of solvent, the reaction was stirred at 40 °C for 18 h. Upon completion of the reaction, as judged by TLC analysis, the crude mixture was purified by flash column chromatography (*n*-hexane/EtOAc, 5:1) to give four diastereomers of the corresponding product **4q** with ratio of 1.4:1.3:1.2:1 (22.7 mg, 41%) as yellow oil; ^1^H-NMR (400 MHz, CDCl_3_) δ 1.13–1.28 (m, 24H), 1.30–1.65 (m, 8H), 1.81–1.91 (m, 2H), 1.95–2.30 (m, 6H), 3.96–4.23 (m, 12H), 4.41 (d, *J* = 4.4 Hz, 1H), 4.44 (d, *J* = 4.4 Hz, 1H), 4.45 (d, *J* = 2.8 Hz, 1H), 4.48 (d, *J* = 2.8 Hz, 1H), 4.63–4.72 (m, 2H), 4.82–4.88 (m, 2H), 7.45–7.49 (m, 8H), 7.56–7.61 (m, 4H), 8.01–8.05 (m, 8H); ^13^C-NMR (100 MHz, CDCl_3_) δ 14.0, 14.0, 21.0, 21.1, 21.2, 29.8, 30.1, 30.5, 30.9, 32.6, 32.6, 33.3, 33.4, 59.6, 59.9, 60.2, 60.6, 61.4, 61.5, 75.2, 75.4, 75.8, 77.2, 77.7, 77.7, 78.0, 128.6, 128.6, 128.7, 128.7, 128.8, 128.8, 128.9, 133.4, 133.7, 136.5, 136.9, 167.6, 167.9, 168.0, 193.6, 193.7; IR (NaCl, neat) ν 2976, 2872, 1734, 1684 cm^−1^; HRMS (ESI) calcd for C_16_H_20_NaO_4_ [M + Na]^+^ 299.1259, found 299.1263.

*Ethyl 3-oxo-3-phenyl-2-(tetrahydro-2H-pyran-2-yl)propanoate* (**4r**) [60]. Tetrahydropyran **1c** (2 mL) was added to PhI=NTs (186 mg, 0.50 mmol) in a 5 mL round-bottomed flask and stirred for 50 min at 65 °C (40% conversion based on ^1^H-NMR analysis). The solvent was removed under reduced pressure and the flask back filled with N_2_(g). To the crude mixture, ethyl benzoylacetate **3a** (69 μL, 0.40 mmol) and DBU (3 μL, 0.025 mmol) were subsequently added. In the absence of solvent, the reaction was stirred at 80 °C for 18 h. Upon completion of the reaction, as judged by TLC analysis, the crude mixture was purified by flash column chromatography (*n*-hexane/EtOAc, 5:1) to give two diastereomers of the corresponding product **4r** with ratio of 1.7:1 (31.2 mg 57%) as yellow oil; ^1^H-NMR (300 MHz, CDCl_3_) δ 1.16–1.20 (m, 6H), 1.40–1.70 (m, 8H), 1.73–1.89 (m, 4H), 3.40–3.46 (m, 1H), 3.48–3.54 (m, 1H), 3.83–3.86 (m, 1H), 3.99–4.02 (m, 1H), 4.09–4.23 (m, 6H), 4.46 (d, *J* = 9.2 Hz, 1H), 4.47 (d, *J* = 9.2 Hz, 1H), 7.44–7.50 (m, 4H), 7.54–7.61 (m, 2H), 8.01–8.05 (m, 4H); ^13^C-NMR (75 MHz, CDCl_3_) δ 14.0, 23.1, 23.2, 25.8, 25.8, 29.7, 29.9, 59.9, 60.7, 61.4, 61.5, 68.8, 68.9, 76.9, 77.1, 128.6, 128.8, 133.3, 133.8, 136.4, 136.6, 137.2, 167.2, 167.8, 192.7, 193.7.

*Diisopropyl 2-(4-hydroxy-1-(4-methylphenylsulfonamido)butyl)malonate* (**5n**). THF **1a** (2 mL) was added to PhI=NTs (186 mg, 0.50 mmol) in a 5 mL round-bottomed flask and stirred for 50 min at room temperature. The solvent was then removed under reduced pressure and the flask back filled with N_2_(g). To the crude mixture, diisopropyl malonate **3n** (0.19 mL, 1.0 mmol) and DBU (8 μL, 0.05 mmol) and PhMe (2 mL) were subsequently added. The reaction was stirred at room temperature for 18 h. Upon completion of the reaction, as judged by TLC analysis, the crude mixture was purified by flash column chromatography (*n*-hexane/EtOAc, 1:1) to give the corresponding product **5n** (50.5 mg, 25%) as white solid; mp 108–114 °C; ^1^H-NMR (400 MHz, CDCl_3_) δ 1.18 (t, *J* = 6.0 Hz, 6H), 1.23 (d, *J* = 6.0 Hz, 6H), 1.37–1.56 (m, 2H), 1.65 (q, *J* = 7.6 Hz, 2H), 2.41 (s, 3H), 3.48 (d, *J* = 4.0 Hz, 1H), 3.51–3.54 (m, 2H), 3.87–3.94 (m, 1H), 4.86–4.92 (m, 1H), 4.99 (m, *J* = 6.0 Hz, 1H), 5.03 (m, *J* = 6.0 Hz, 1H), 5.71 (d, *J* = 9.6 Hz, 1H), 7.28 (d, *J* = 8.0 Hz, 2H), 7.75 (d, *J* = 8.0 Hz, 2H); ^13^C-NMR (100 MHz, CDCl_3_) δ 21.4, 21.5, 21.5, 21.6, 21.6, 28.7, 29.6, 53.1, 55.2, 61.8, 69.4, 69.7, 127.0, 129.6, 138.4, 143.3, 167.0, 167.6; IR (NaCl, neat) ν 3362, 3310, 2983, 2936, 1732, 1722, 1599 cm^−1^; HRMS (ESI) calcd for C_20_H_32_NO_7_S [M + H]^+^ 430.1899, found 430.1892.

## 4. Conclusions

In summary, a mild transition metal-free cross dehydrogenative coupling (CDC) synthetic route to 2-tetrahydrofuran and –pyran substituted 1,3-carbonyl compounds from commercially available cyclic ethers and 1,3-dicarbonyl derivatives has been developed. Achieved in moderate to excellent yields of 32%–80%, the synthetic method was shown to tolerate β-ketoesters, dialkyl malonates and 1,3-diones, which complements and supplements the existing transition metal approaches. The present method also shows the promising utility of other hypervalent iodine reagents other than diaryliodonium salts for transition metal-free CDC reactions. Further exploration on the utility of iminoiodanes is currently underway.

## Supplementary Materials

Supplementary materials can be accessed at: http://www.mdpi.com/1420-3049/20/07/13336/s1.

## References

[B1-molecules-20-13336] 1.For recent reviews on green and sustainable chemistry, see refs. 2–4.

[2] Gaich T., Baran P.S. (2010). Aiming for the ideal synthesis. J. Org. Chem..

[3] Li C.J., Trost B.M. (2008). Green chemistry for chemical synthesis. Proc. Natl. Acad. Sci. USA.

[4] Constable D.J.C., Curzons A.D., Cunningham V.L. (2002). Metrics to “green chemistry”—Which are the best?. Green Chem..

[B5-molecules-20-13336] 5.For recent reviews on transition metal-catalyzed cross dehydrogenative coupling (CDC) for C–C bond formation, see refs. 6–9.

[6] Tsurugi H., Yamamoto K., Nagae H., Kaneko H., Mashima K. (2014). Direct functionalization of unactivated C–H bonds catalyzed by group 3–5 metal alkyl complexes. Dalton Trans..

[7] Girard S.A., Knauber T., Li C.J. (2014). The cross-dehydrogenative coupling of Csp^3^–H bonds: A versatile strategy for C–C bond formations. Angew. Chem. Int. Ed..

[8] Cho S.H., Kim J.Y., Kwak J., Chang S. (2011). Recent advances in the transition metal-catalyzed twofold oxidative C–H bond activation strategy for C–C and C–N bond formation. Chem. Soc. Rev..

[9] Yeung C.S., Dong V.M. (2011). Catalytic dehydrogenative cross-coupling: Forming carbon-carbon bonds by oxidizing two carbon-hydrogen bonds. Chem. Rev..

[B10-molecules-20-13336] 10.For selected examples of Pd-catalyzed CDC for C–C bond formation, see refs. 11–23.

[11] Szabo F., Simko D., Novak Z. (2014). A one-pot process for palladium catalyzed direct C–H acylation of anilines in water using a removable ortho directing group. RSC. Adv..

[12] Jafarpour F., Hazrati H., Mohasselyazdi N., Khoobi M., Shafiee A. (2013). Palladium catalyzed dehydrogenative arylation of coumarins: An unexpected switch in regioselectivity. Chem. Commun..

[13] Shang Y., Jie X., Zhou J., Hu P., Huang S., Su W. (2013). Pd-Catalyzed C–H olefination of (hetero)arenes by using saturated ketones as an olefin source. Angew. Chem. Int. Ed..

[14] Bugaut X., Glorius F. (2011). Palladium-catalyzed selective dehydrogenative cross-couplings of heteroarenes. Angew. Chem. Int. Ed..

[15] Li H., Liu J., Sun C.L., Li B.J., Shi Z.J. (2011). Palladium-catalyzed cross-coupling of polyfluoroarenes with simple arenes. Org. Lett..

[16] Zhao X., Yeung C.S., Dong V.M. (2010). Palladium-catalyzed ortho-arylation of *o*-phenylcarbamates with simple arenes and sodium persulfate. J. Am. Chem. Soc..

[17] Wasa M., Engle K.M., Yu J.Q. (2010). Pd(II)-Catalyzed Olefination of sp^3^ C–H Bonds. J. Am. Chem. Soc..

[18] Wang D.H., Engle K.M., Shi B.F., Yu J.Q. (2010). Ligand-enabled reactivity and selectivity in a synthetically versatile aryl C–H olefination. Science.

[19] Wu J., Cui X., Chen L., Jiang G., Wu Y. (2009). Palladium-catalyzed alkenylation of quinoline-*N*-oxides via C–H activation under external-oxidant-free conditions. J. Am. Chem. Soc..

[20] Li J.J., Mei T.S., Yu J.Q. (2008). Synthesis of indolines and tetrahydroisoquinolines from arylethylamines by PdII-catalyzed C–H activation reactions. Angew. Chem. Int. Ed..

[21] Houlden C.E., Bailey C.D., Gair Ford J., Gagné M.R., Lloyd-Jones G.C., Booker-Milburn K.I. (2008). Distinct reactivity of Pd(OTs)_2_: The intermolecular Pd(II)-catalyzed 1,2-carboamination of dienes. J. Am. Chem. Soc..

[22] Li B.J., Tian S.L., Fang Z., Shi Z.J. (2008). Multiple C–H activations to construct biologically active molecules in a process completely free of organohalogen and organometallic components. Angew. Chem. Int. Ed..

[23] Hull K.L., Sanford M.S. (2007). Catalytic and highly regioselective cross-coupling of aromatic C–H substrates. J. Am. Chem. Soc..

[B24-molecules-20-13336] 24.For selected examples on CDC C–C bond formation catalyzed by other transition metal, see refs. 25–35.

[25] Shang M., Wang H.L., Sun S.Z., Dai H.X., Yu J.Q. (2014). Cu(II)-mediated ortho C–H alkynylation of (hetero)arenes with terminal alkynes. J. Am. Chem. Soc..

[26] Vora H.U., Silvestri A.P., Engelin C.J., Yu J.Q. (2014). Rhodium(II)-catalyzed nondirected oxidative alkenylation of arenes: Arene loading at one equivalent. Angew. Chem. Int. Ed..

[27] Odani R., Hirano K., Satoh T., Miura M. (2013). Copper-mediated dehydrogenative biaryl coupling of naphthylamines and 1,3-azoles. J. Org. Chem..

[28] Pan F., Lei Z.Q., Wang H., Li H., Sun J., Shi Z.J. (2013). Rhodium(I)-catalyzed redox-economic cross-coupling of carboxylic acids with arenes directed by *N*-containing groups. Angew. Chem. Int. Ed..

[29] Qin X., Liu H., Qin D., Wu Q., You J., Zhao D., Guo Q., Huang X., Lan J. (2013). Chelation-assisted Rh(III)-catalyzed C2-selective oxidative C–H/C–H cross-coupling of indoles/pyrroles with arenes. Chem. Sci..

[30] Hirano K., Miura M. (2012). Copper-mediated oxidative direct C–C (hetero)aromatic cross-coupling. Chem. Commun..

[31] Wencel-Delord J., Nimphius C., Patureau F.W., Glorius F. (2012). [RhIIICp*]-Catalyzed dehydrogenative aryl-aryl bond formation. Angew. Chem. Int. Ed..

[32] Wang F., Song G.Y., Li X.W. (2010). Rh(III)-Catalyzed tandem oxidative olefination-michael reactions between aryl carboxamides and alkenes. Org. Lett..

[33] Patureau F.W., Glorius F. (2010). Rh Catalyzed olefination and vinylation of unactivated acetanilides. J. Am. Chem. Soc..

[34] Deng G., Zhao L., Li C.J. (2008). Ruthenium-catalyzed oxidative cross-coupling of chelating arenes and cycloalkanes. Angew. Chem. Int. Ed..

[35] Ueura K., Satoh T., Miura M. (2007). An efficient waste-free oxidative coupling via regioselective C–H bond cleavage: Rh/Cu-catalyzed reaction of benzoic acids with alkynes and acrylates under air. Org. Lett..

[B36-molecules-20-13336] 36.For selected examples on transition metal-catalyzed CDC C–C bond formation of amines, see refs. 37–46.

[37] Jin X., Yamaguchi K., Mizuno N. (2014). Aerobic cross-dehydrogenative coupling of terminal alkynes and tertiary amines by a combined catalyst of Zn^2+^ and OMS-2. RSC. Adv..

[38] Zhong J.J., Meng Q.Y., Liu B., Li X.B., Gao X.W., Lei T., Wu C.J., Li Z.J., Tung C.H., Wu L.Z. (2014). Cross-coupling hydrogen evolution reaction in homogeneous solution without noble metals. Org. Lett..

[39] Li G., Qian S., Wang C., You J. (2013). Palladium(II)-catalyzed dehydrogenative cross-coupling between two Csp^3^–H bonds: Unexpected C=C bond formation. Angew. Chem. Int. Ed..

[40] Nie S.Z., Sun X., Wei W.T., Zhang X.J., Yan M., Xiao J.L. (2013). Unprecedented construction of C=C double bonds via Ir-catalyzed dehydrogenative and dehydrative cross-couplings. Org. Lett..

[41] Alagiri K., Prabhu K.R. (2012). C–H functionalization of tertiary amines by cross dehydrogenative coupling reactions: Solvent-free synthesis of α-iminonitriles and β-nitroamines under aerobic condition. Org. Biomol. Chem..

[42] Xie J., Li H., Zhou J., Cheng Y., Zhu C. (2012). A Highly efficient gold-catalyzed oxidative C–C coupling from C–H bonds using air as oxidant. Angew. Chem. Int. Ed..

[43] Huang L., Niu T., Wu J., Zhang Y. (2011). Copper-catalyzed oxidative cross-coupling of *N*,*N*-dimethylanilines with heteroarenes with molecular oxygen. J. Org. Chem..

[44] Shirakawa E., Uchiyama N., Hayashi T. (2011). Iron-catalyzed oxidative coupling of alkylamides with arenes through oxidation of alkylamides followed by Friedel-crafts alkylation. J. Org. Chem..

[45] Yang F., Li J., Xie J., Huang Z.Z. (2010). Copper-catalyzed dehydrogenative coupling reactions of tertiary amines with ketones or indoles. Org. Lett..

[46] Liu P., Zhou C.Y., Xiang S., Che C.M. (2010). Highly efficient oxidative carbon–carbon coupling with SBA-15-support iron terpyridine catalyst. Chem. Commun..

[B47-molecules-20-13336] 47.For selected examples on transition metal-catalyzed CDC of ethers for C–C bond formation, see refs. 48–61.

[48] Pandit R.P., Lee Y.R. (2014). Direct oxidative arylation of C(sp^3^)–H bonds adjacent to oxygen of ethers and alcohols. Adv. Synth. Catal..

[49] Zhou L., Tang S., Qi X., Lin C., Lin K., Liu C., Lan Y., Lei A. (2014). Transition-metal-assisted radical/radical cross-coupling: A new strategy to the oxidative C(sp^3^)−H/N−H cross-coupling. Org. Lett..

[50] Rout S.K., Guin S., Ali W., Gogoi A., Patel B.K. (2014). Copper-catalyzed esterification of alkylbenzenes with cyclic ethers and cycloalkanes via C(sp^3^)–H activation following cross-dehydrogenative coupling. Org. Lett..

[51] Siddaraju Y., Lamani M., Prabhu K.R. (2014). A Transition metal-free minisci reaction: Acylation of isoquinolines, quinolines, and quinoxaline. J. Org. Chem..

[52] Liu D., Liu C., Li H., Lei A. (2014). Copper-catalyzed oxidative C–H/C–H coupling between olefins and simple ethers. Chem. Commun..

[53] Wu Z., Pi C., Cui X., Bai J., Wu Y. (2013). Direct C-2 alkylation of quinoline *N*-oxides with ethers via palladium-catalyzed dehydrogenative cross-coupling reaction. Adv. Synth. Catal..

[54] Xie Z., Cai Y., Hu H., Lin C., Jiang J., Chen Z., Wang L., Pan Y. (2013). Cu-catalyzed cross-dehydrogenative coupling reaction of (benzo)thiazoles with cyclic ethers. Org. Lett..

[55] Liu D., Liu C., Li H., Lei A. (2013). Direct functionalization of tetrahydrofuran and 1,4-dioxane: Nickel-catalyzed oxidative C(sp^3^)−H arylation. Angew. Chem. Int. Ed..

[56] Park S.J., Price J.R., Todd M.H. (2012). Oxidative arylation of isochroman. J. Org. Chem..

[57] Kumar G.S., Pieber B., Reddy R., Oliver Kappe C. (2012). Copper-catalyzed formation of C–O bonds by direct α-C–H bond activation of ethers using stoichiometric amounts of peroxide in batch and continuous-flow formats. Chem. Eur. J..

[58] Cui Z., Shang X., Shao X.F., Liu Z.Q. (2012). Copper-catalyzed decarboxylative alkenylation of sp^3^ C–H bonds with cinnamic acids via a radical process. Chem. Sci..

[59] Guo X., Pan S., Liu J., Li Z. (2009). One-pot synthesis of symmetric and unsymmetric 1,1-bis-indolylmethanes via tandem iron-catalyzed C–H bond oxidation and C–O bond cleavage. J. Org. Chem..

[60] Li Z., Yu R., Li H. (2008). Iron-catalyzed C–C bond formation by direct functionalization of C–H bonds adjacent to heteroatoms. Angew. Chem. Int. Ed..

[61] Zhang Y., Li C.J. (2006). Highly efficient cross-dehydrogenative-coupling between ethers and active methylene compounds. Angew. Chem. Int. Ed..

[B62-molecules-20-13336] 62.For reviews on transition metal-free CDC, see refs. 63 and 64.

[63] Sun C.L., Shi Z.J. (2014). Transition-metal-free coupling reactions. Chem. Rev..

[64] Klussmann M., Sureshkumar D. (2011). Catalytic oxidative coupling reactions for the formation of carbon–carbon bonds without carbon–metal intermediates. Synthesis.

[B65-molecules-20-13336] 65.For selected examples concerning transition metal-free CDC, see refs. 66–71.

[66] Tanoue A., Yoo W.J., Kobayashi S. (2014). Sulfuryl chloride as an efficient initiator for the metal-free aerobic cross-dehydrogenative coupling reaction of tertiary amines. Org. Lett..

[67] Dhineshkumar J., Lamani M., Alagiri K., Prabhu K.R. (2013). A versatile C–H functionalization of tetrahydroisoquinolines catalyzed by iodine at aerobic conditions. Org. Lett..

[68] Schweitzer-Chaput B., Klussmann M. (2013). Brønsted acid catalyzed C–H functionalization of *N*-protected tetrahydroisoquinolines via intermediate peroxides. Eur. J. Org. Chem..

[69] Kumar R.A., Saidulu G., Prasad K.R., Kumar G.S., Sridhar B., Reddy K.R. (2012). Transition metal-free α-C(sp^3^)–H bond functionalization of amines by oxidative cross dehydrogenative coupling reaction. Simple and direct access to *C*-4-alkylated 3,4-dihydroquinazoline derivatives. Adv. Synth. Catal..

[70] Richter H., Froehlich R., Daniliuc C.G., Garcia Mancheño O. (2012). Mild metal-free tandem α-alkylation/cyclization of *N*-benzyl carbamates with simple olefins. Angew. Chem. Int. Ed..

[71] Alagiri K., Devadig P., Prabhu K.R. (2012). CDC reactions of *N*-aryl tetrahydroisoquinolines using catalytic amounts of DDQ: C–H activation under aerobic conditions. Chem. Eur. J..

[72] Merritt E.A., Olofsson B. (2009). Diaryliodonium Salts: A Journey from Obscurity to Fame. Angew. Chem. Int. Ed..

[B73-molecules-20-13336] 73.For selected examples on transition metal-free CDC using hypervalent iodine, see refs. 74–81.

[74] Narayan R., Antonchick A.P. (2014). Hypervalent iodine-mediated selective oxidative functionalization of (thio)chromones with alkanes. Chem. Eur. J..

[75] Antonchick A.P., Burgmann L. (2013). Direct selective oxidative cross-coupling of simple alkanes with heteroarenes. Angew. Chem. Int. Ed..

[76] Matcha K., Antonchick A.P. (2013). Metal-free cross-dehydrogenative coupling of heterocycles with aldehydes. Angew. Chem. Int. Ed..

[77] Castro S., Fernandez J.J., Vicente R., Fananas F.J., Rodriguez F. (2012). Base- and metal-free C–H direct arylations of naphthalene and other unbiased arenes with diaryliodonium salts. Chem. Commun..

[78] Wen J., Zhang R.Y., Chen S.Y., Zhang J., Yu X.Q. (2012). Direct arylation of arene and *N*-heteroarenes with diaryliodonium salts without the use of transition metal catalyst. J. Org. Chem..

[79] Ackermann L., Dell’Acqua M., Fenner S., Vicente R., Sandmann R. (2011). Metal-free direct arylations of indoles and pyrroles with diaryliodonium Salts. Org. Lett..

[80] Kita Y., Morimoto K., Ito M., Ogawa C., Goto A., Dohi T. (2009). Metal-free oxidative cross-coupling of unfunctionalized aromatic compounds. J. Am. Chem. Soc..

[81] Dohi T., Ito M., Morimoto K., Iwata M., Kita Y. (2008). Oxidative cross-coupling of arenes induced by single-electron transfer leading to biaryls using organoiodine(III) oxidants. Angew. Chem. Int. Ed..

[B82-molecules-20-13336] 82.Selected general reviews on transition-metal-mediated imido/nitrene reactions, see refs. 83–91

[83] Dequirez G., Pons V., Dauban P. (2012). Nitrene chemistry in organic synthesis: Still in its infancy?. Angew. Chem. Int. Ed..

[84] Roizen J.L., Harvey M.E., Du Bois (2012). Metal-catalyzed nitrogen-atom transfer methods for the oxidation of aliphatic C–H bonds. Acc. Chem. Res..

[85] Collet F., Lescot C., Dauban P. (2011). Catalytic C-H amination: The stereoselectivity issue. Chem. Soc. Rev..

[86] Chang J.W.W., Ton T.M.U., Chan P.W.H. (2011). Transition-metal-catalyzed aminations and aziridinations of C–H and C=C bonds with iminoiodinane. Chem. Rec..

[87] Collet F., Dodd R.H., Dauban P. (2009). Catalytic C–H amination: Recent progress and future directions. Chem. Commun..

[88] Díaz-Requejo M.M., Pérez P.J. (2008). Coinage Metal catalyzed C–H bond functionalization of hydrocarbons. Chem. Rev..

[89] Davies H.M. L., Manning J.R. (2008). Catalytic C–H functionalization by metal carbenoid and nitrenoid insertion. Nature.

[90] Davies H.M.L. (2006). Recent advances in catalytic enantioselective intermolecular C–H functionalization. Angew. Chem. Int. Ed..

[91] Müller P., Fruit C. (2003). Enantioselective catalytic aziridinations and asymmetric nitrene insertions into C–H bonds. Chem. Rev..

[B92-molecules-20-13336] 92.For selected recent examples on transition-metal-free reactions with nitrenoid precursors, see refs. 93–103.

[93] Kiyokawa K., Kosaka T., Minakata S. (2013). Metal-free aziridination of styrene derivatives with iminoiodinane catalyzed by a combination of iodine and ammonium iodide. Org. Lett..

[94] Souto J.A., Martínez C., Velilla I., Muñiz K. (2013). Defined hypervalent iodine(III) regents incorporating transferable nitrogen groups: Nucleophilic amination through electrophilic activation. Angew. Chem. Int. Ed..

[95] Ochiai M., Yamane S., Hoque M.M., Saito M., Miyamoto K. (2012). Metal-free α-CH amination of ethers with hypervalent sulfonylimino-λ^3^-bromane that acts as an active nitrenoid. Chem. Commun..

[96] Ochiai M., Miyamoto T., Kaneaki K., Hayashi S., Nakanishi W. (2011). Highly regioselective amination of unactivated alkanes by hypervalent sulfonylimino-λ^3^-bromane. Science.

[97] Takeda Y., Hayakawa J., Yano K., Minakata S. (2012). Transition-metal-free benzylic C–H bond intermolecular amination utilizing chloramine-T and I_2_. Chem. Lett..

[98] Zhang D.H., Wei Y., Shi M. (2011). Metal-free ring expansions of methylenecyclopropanes through nitrene equivalent. Eur. J. Org. Chem..

[99] Karabal P.U., Chouthaiwale P.V., Shaikh T.M., Suryavanshi G., Sudalai A. (2010). NIO_4_/LiBr-mediated aziridination of olefins using chloramine-T. Tetrahedron Lett..

[100] Fang C., Qian W., Bao W. (2008). A mild and clean method for oxidative formation of amides from aldehydes and amines. Synlett.

[101] Li J., Chan P.W.H., Che C.M. (2005). Aryl iodide mediated aziridination of alkenes. Org. Lett..

[102] Lim B.W., Ahn K.H. (1996). The reaction of [*N*-(*p*-toluenesulfonyl)lmino]-phenyliodinane with enol silanes. Synth. Commun..

[103] Tejo C., Yeo H.Q., Chan P.W.H. (2014). Brønsted acid catalyzed amination of 1,3-dicarbonyl compounds by iminoiodanes. Synlett.

[B104-molecules-20-13336] 104.For selected recent works by our group, see refs. 103 and 105–109.

[105] Ton T.M.U., Himawan F., Chang J.W.W., Chan P.W.H. (2012). Copper(II) triflate catalyzed amination of 1,3-dicarbonyl compounds. Chem. Eur. J..

[106] Ton T.M.U., Tejo C., Tiong D.L.Y., Chan P.W.H. (2012). Copper(II) triflate catalyzed amination and aziridination of 2-alkyl substituted 1,3-dicarbonyl compounds. J. Am. Chem. Soc..

[107] Ton T.M.U., Tejo C., Tania S., Chang J.W.W., Chan P.W.H. (2011). Iron(III)-catalyzed amidation of aldehydes with iminoiodanes at room temperature and under microwave-assisted conditions. J. Org. Chem..

[108] Chang J.W.W., Ton T.M.U., Tania S., Taylor P.C., Chan P.W.H. (2010). Practical copper(I)-catalyzed amidation of aldehydes. Chem. Commun..

[109] Chang J.W.W., Chan P.W.H. (2008). Highly Efficient Ruthenium(II) Porphyrin-Catalyzed Amidation of Aldehydes. Angew. Chem. Int. Ed..

[B110-molecules-20-13336] 110.For reports correlating 1,3-dicarbonyl compound reactivity to pKa values, see refs. 103, 111 and 112.

[111] Arnett E.M., Maroldo S.G., Schilling S.L., Harrelson J.A. (1984). Ion pairing and reactivity of enolate anions. 5. Thermodynamics of ionization of β-di- and tricarbonyl compounds in dimethyl sulfoxide solution and ion pairing of their alkali salts. J. Am. Chem. Soc..

[112] Olmstead W.N., Bordwell F.G. (1980). Ion-pair association constants in dimethyl sulfoxide. J. Org. Chem..

[B113-molecules-20-13336] 113.Treatment of **5n** under the optimized conditions at room temperature led to an observed 6% conversion to **2a** and **3n**. For selected studies on retro-Aldol or retro-Mannich reactions, see refs. 114–118.

[114] Gao B., Zhao Y., Hu M., Ni C., Hu J. (2014). *gem*-Difluoroolefination of diaryl ketones and enolizable aldehydes with difluoromethyl 2-pyridyl sulfone. New insights into the Julia-Kocienski reaction. Chem. Eur. J..

[115] Bartrum H.E., Viceriat A., Carret S., Poisson J.F. (2014). Sulfinylimidates as chiral amide equivalents for irreversible, asymmetric aldol reactions. Org. Lett..

[116] Uematsu R., Maeda S., Taketsugu T. (2014). Multiple reaction pathways operating in the mechanism of vinylogous Mannich-type reaction activated by a water molecule. Chem. Asian J..

[117] Schmitt D.C., Malow E.J., Johnson J.S. (2012). Three-component glycolate michael reactions of enolates, silyl glyoxylates, and α,β-enones. J. Org. Chem.

[118] Roy S., Davydova M.P., Pal R., Gilmore K., Tolstikov G.A., Vasilevsky S.F., Alabugin I.V. (2011). Dissecting alkynes: Full cleavage of polarized C≡C moiety via sequential bis-Michael Addition/Retro-Mannich cascade. J. Org. Chem..

[119] CCDC 1059668 contains the supplementary crystallographic data for this paper. This data can be obtained free of charge from The Cambridge Crystallographic Data Centre. http://www.ccdc.cam.ac.uk/data_request/cif.

[120] Yamada Y., Yamamoto T., Okawara M. (1975). Synthesis and reaction of new type iodine-nitrogen ylide, *N*-tosyliminoiodane. Chem. Lett..

[121] De Godoy L.A.F., Camilo N.S., Pilli R.A. (2006). Addition of carbon nucleophiles to cyclic *N*-acyliminium and oxocarbenium ions under solvent-free conditions. Tetrahedron Lett..

