# Supplementary material for: Iminoiodane- and Brønsted Base-Mediated Cross Dehydrogenative Coupling of Cyclic Ethers with 1,3-Dicarbonyl Compounds
Source: Molecules. 2015 Jul 22;20(7):13336–53. doi: 10.3390/molecules200713336 (PMC6332110; doi:10.3390/molecules200713336)
Supplement: Supplementary file 1 [file molecules-20-13336-s001.pdf]

Supplementary Materials

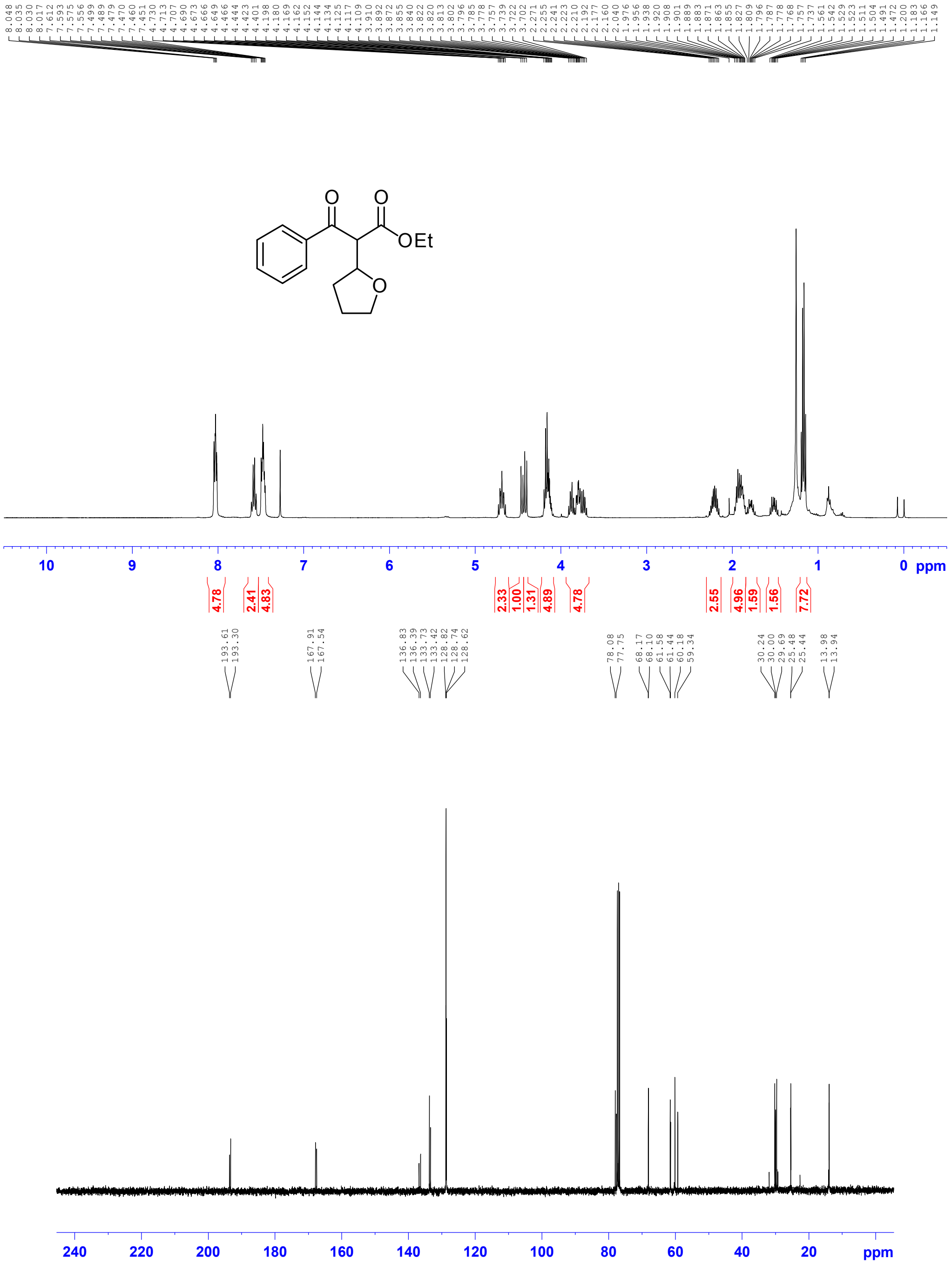

Figure S1. ^{1}H and ^{13}C NMR spectra of ethyl 3-oxo-3-phenyl-2-(tetrahydrofuran-2-yl) propanoate (**4a**).

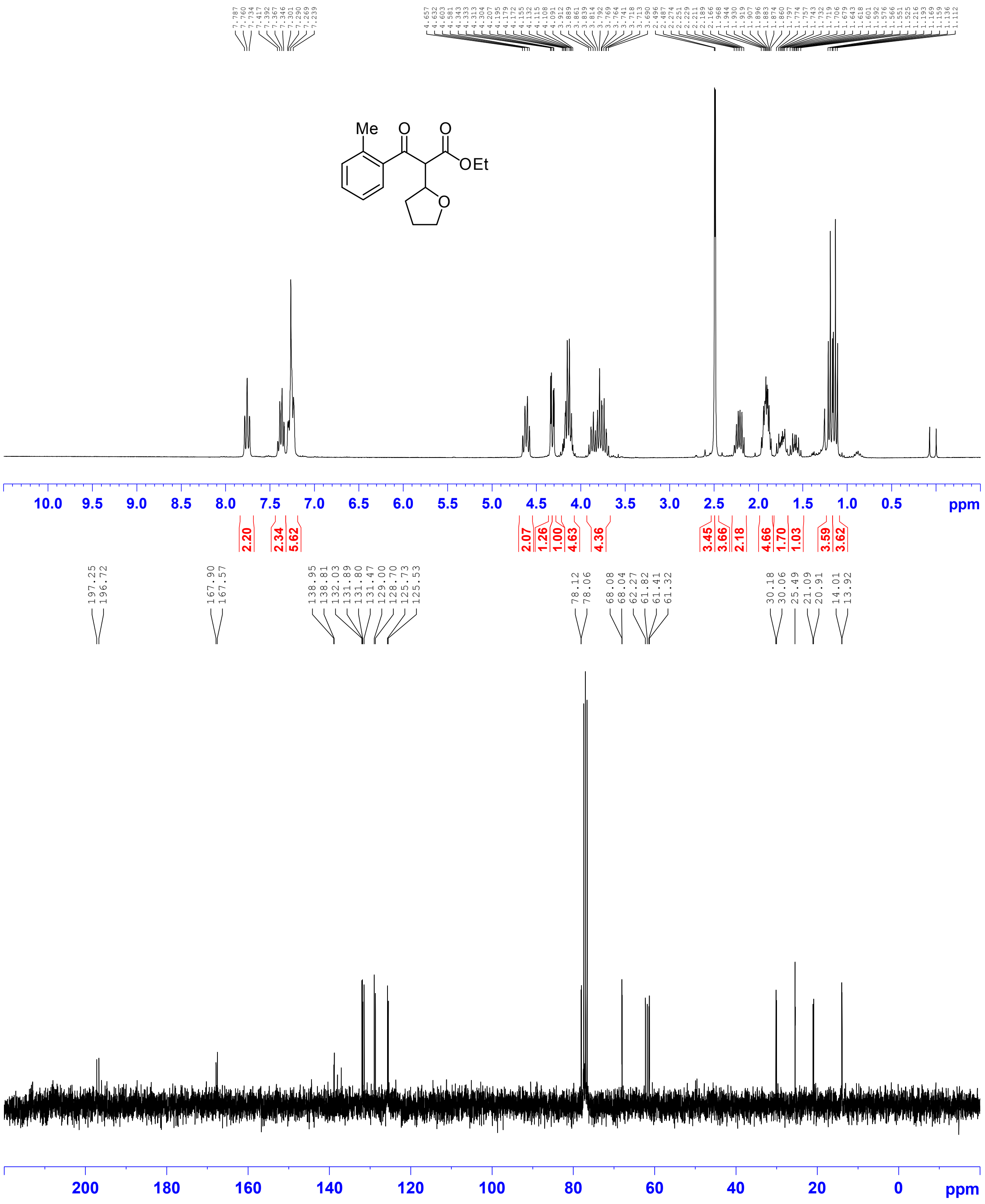

Figure S2. ^{1}H and ^{13}C NMR spectra of ethyl 3-oxo-2-(tetrahydrofuran-2-yl)-3-(o-tolyl) propanoate (**4b**).

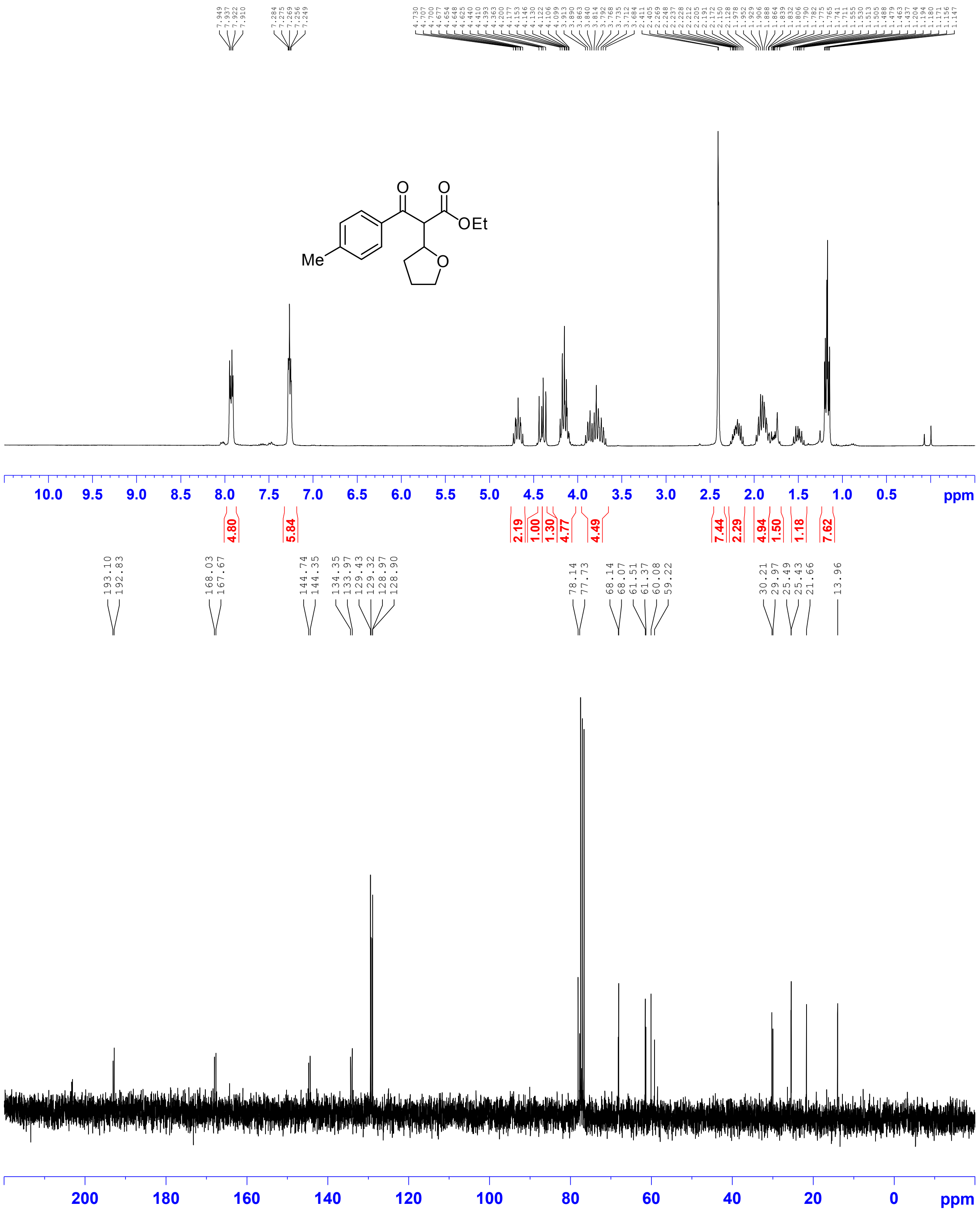

Figure S3. ^{1}H and ^{13}C NMR spectra of ethyl 3-oxo-2-(tetrahydrofuran-2-yl)-3-(p-tolyl) propanoate (**4c**).

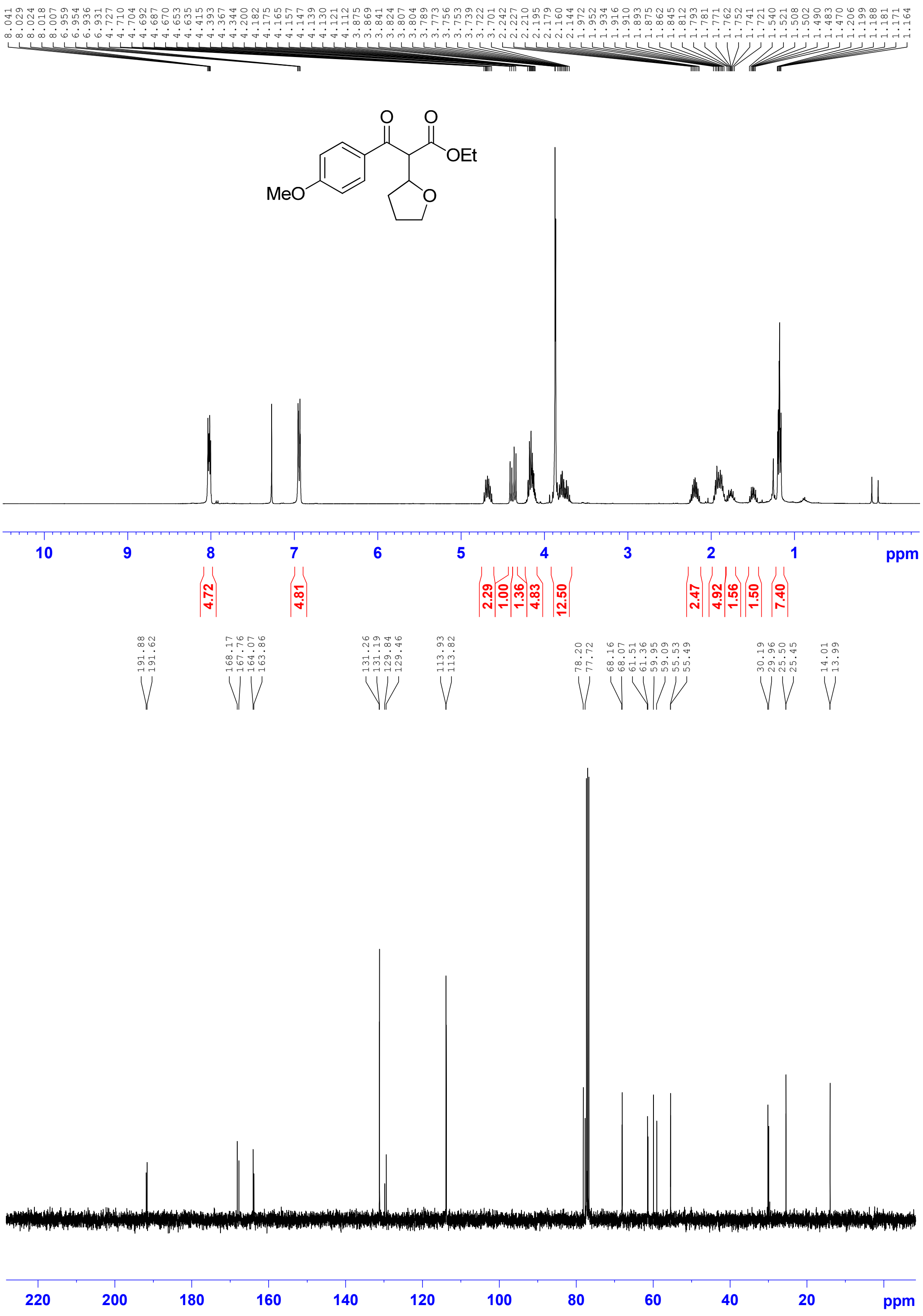

Figure S4. ^{1}H and ^{13}C NMR spectra of ethyl 3-(4-methoxyphenyl)-3-oxo-2-(tetrahydrofuran-2-yl)propanoate (**4d**).

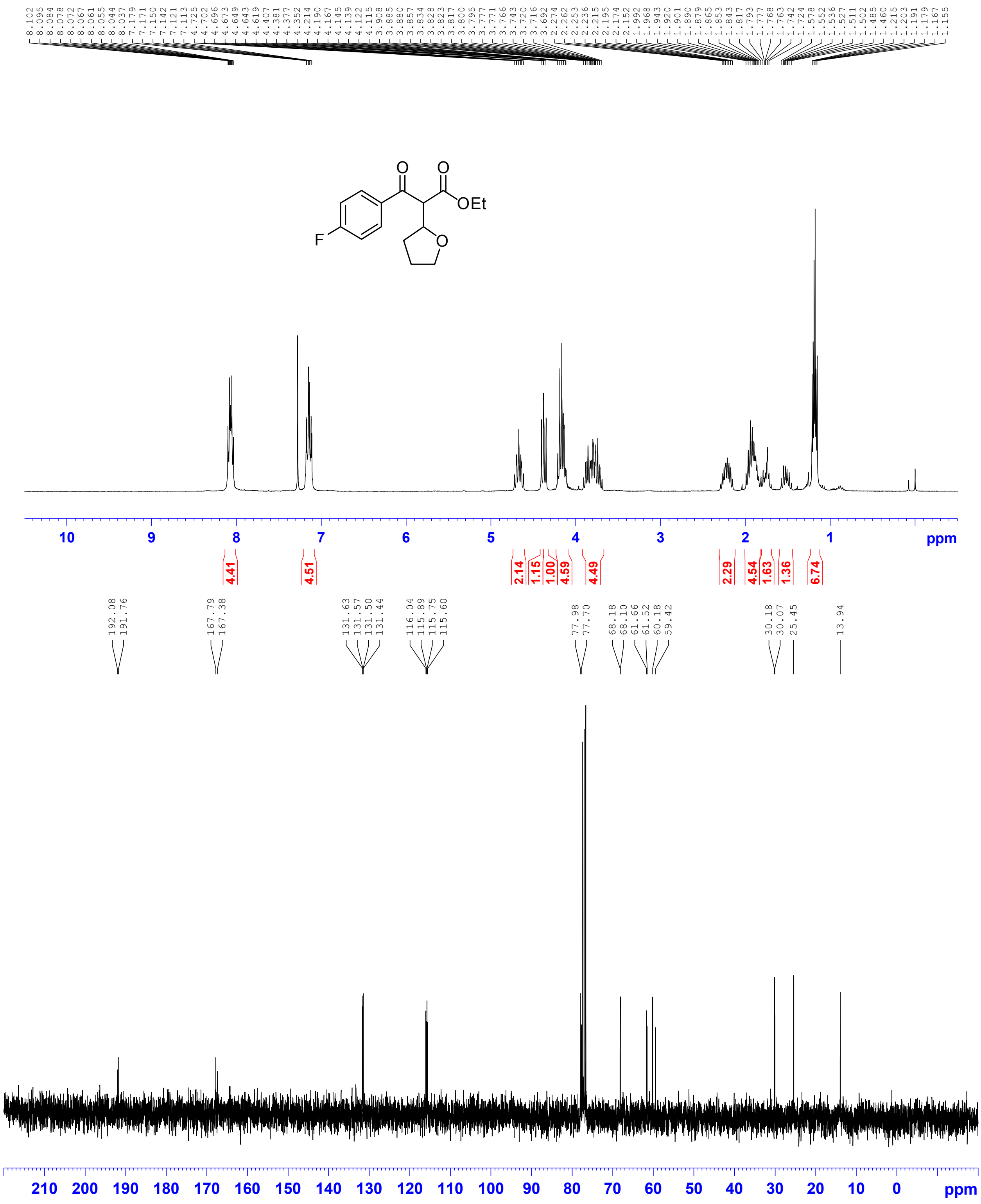

Figure S5. ^{1}H and ^{13}C NMR spectra of ethyl 3-(4-fluorophenyl)-3-oxo-2-(tetrahydrofuran-2-yl)propanoate (**4e**).

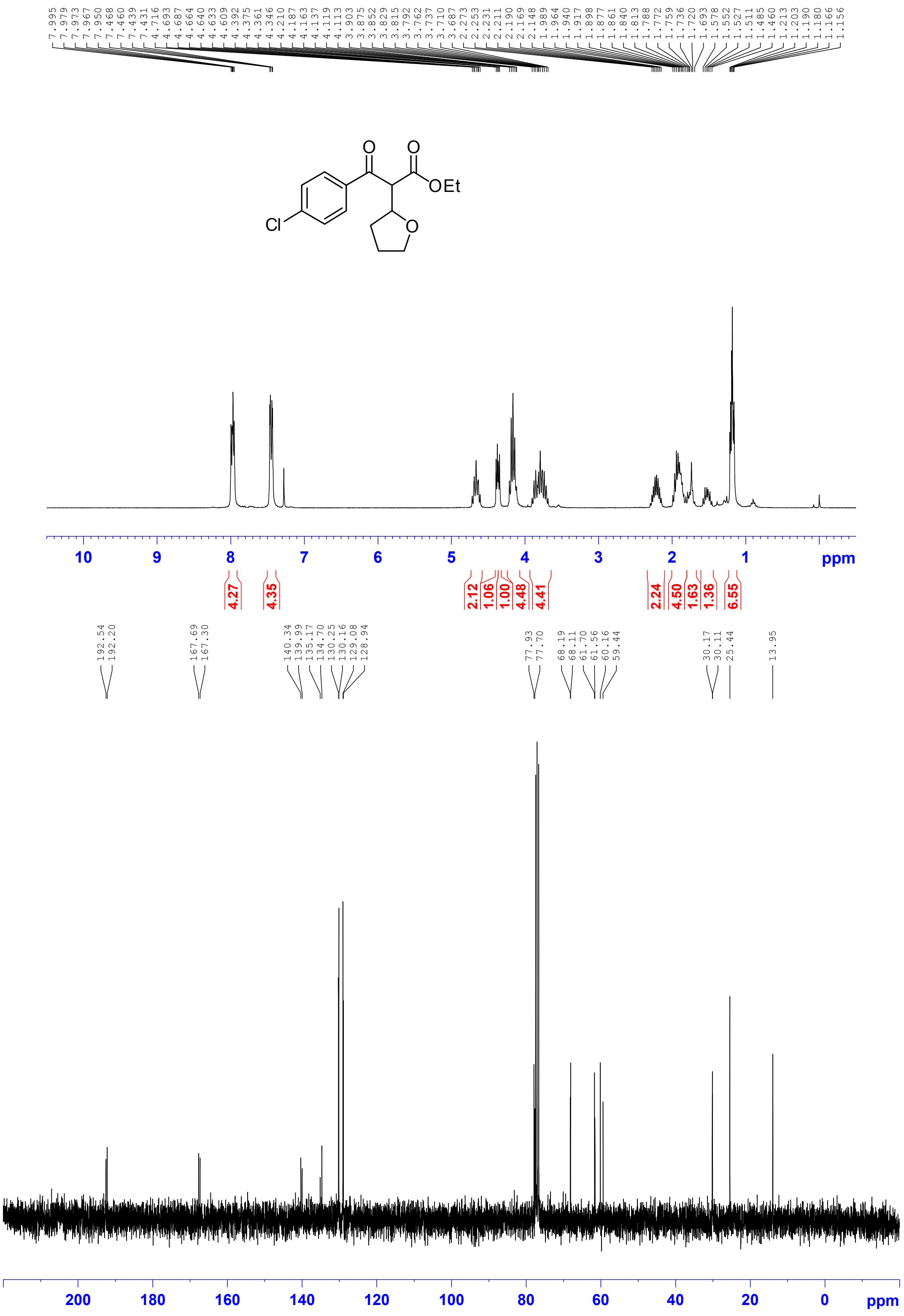

Figure S6. ^{1}H and ^{13}C NMR spectra of ethyl 3-(4-chlorophenyl)-3-oxo-2-(tetrahydrofuran-2-yl)propanoate (**4f**).

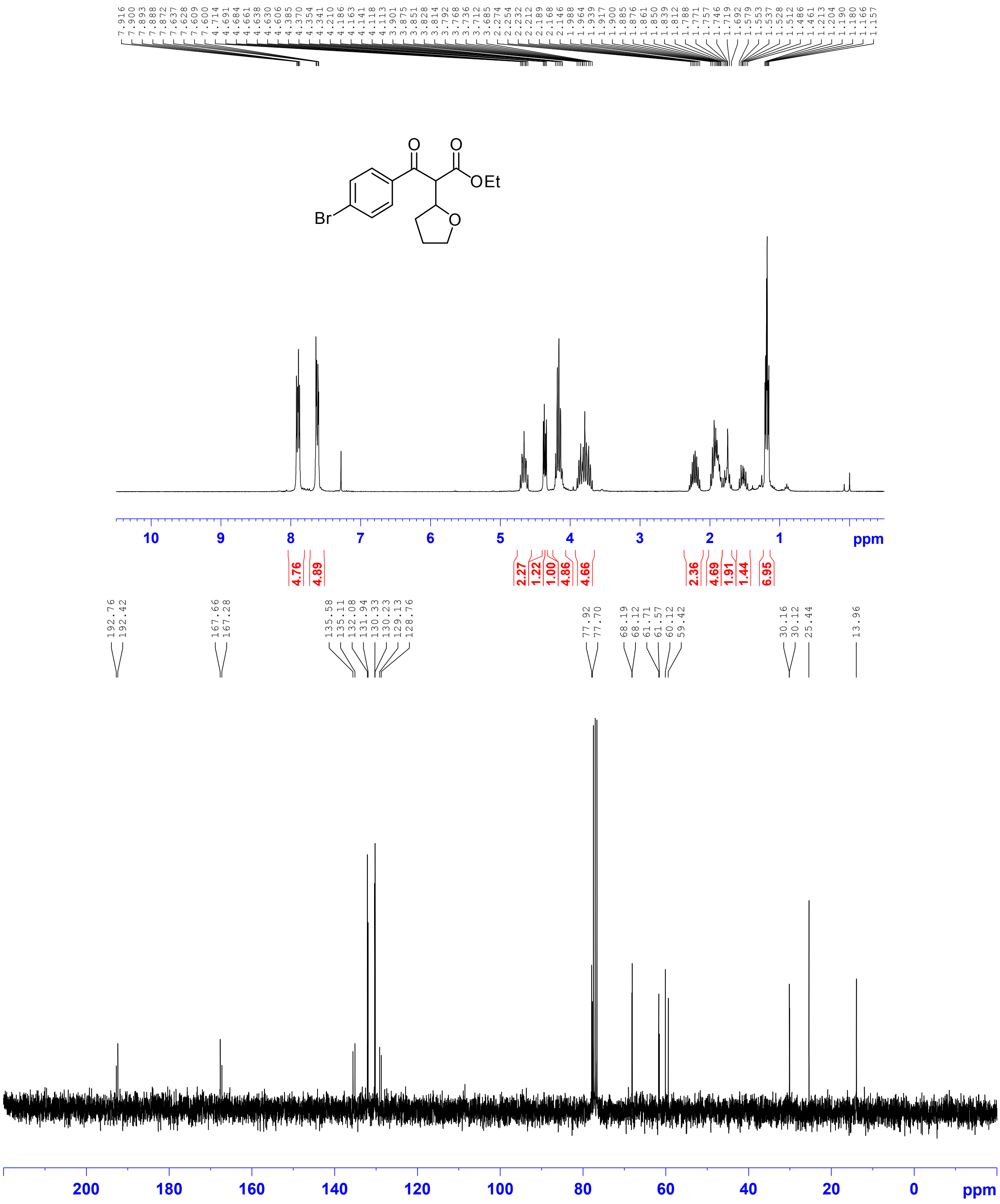

Figure S7. ^{1}H and ^{13}C NMR spectra of ethyl 3-(4-bromophenyl)-3-oxo-2-(tetrahydrofuran-2-yl)propanoate (**4g**).

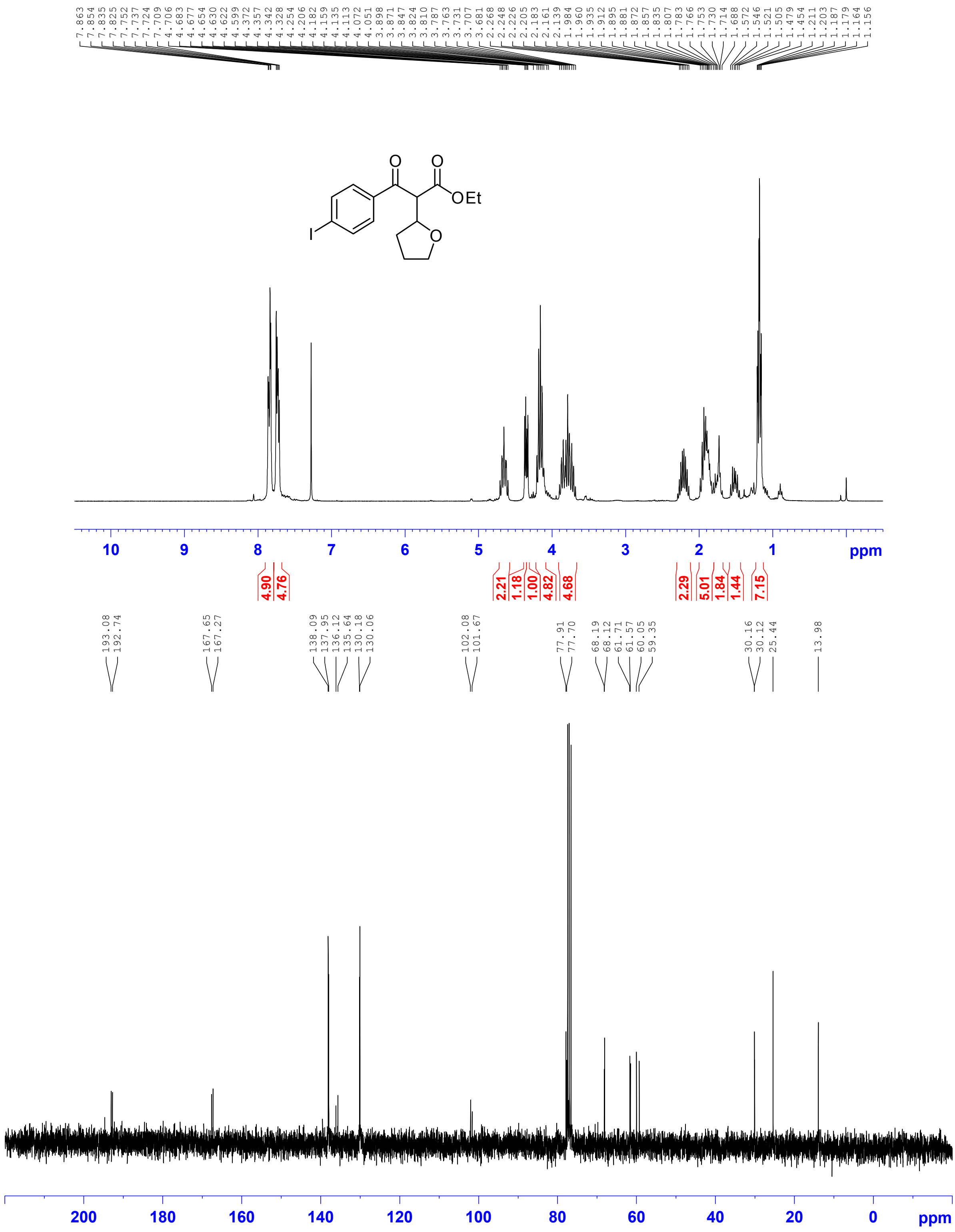

Figure S8. ^{1}H and ^{13}C NMR spectra of ethyl 3-(4-iodophenyl)-3-oxo-2-(tetrahydrofuran-2-yl) propanoate (**4h**).

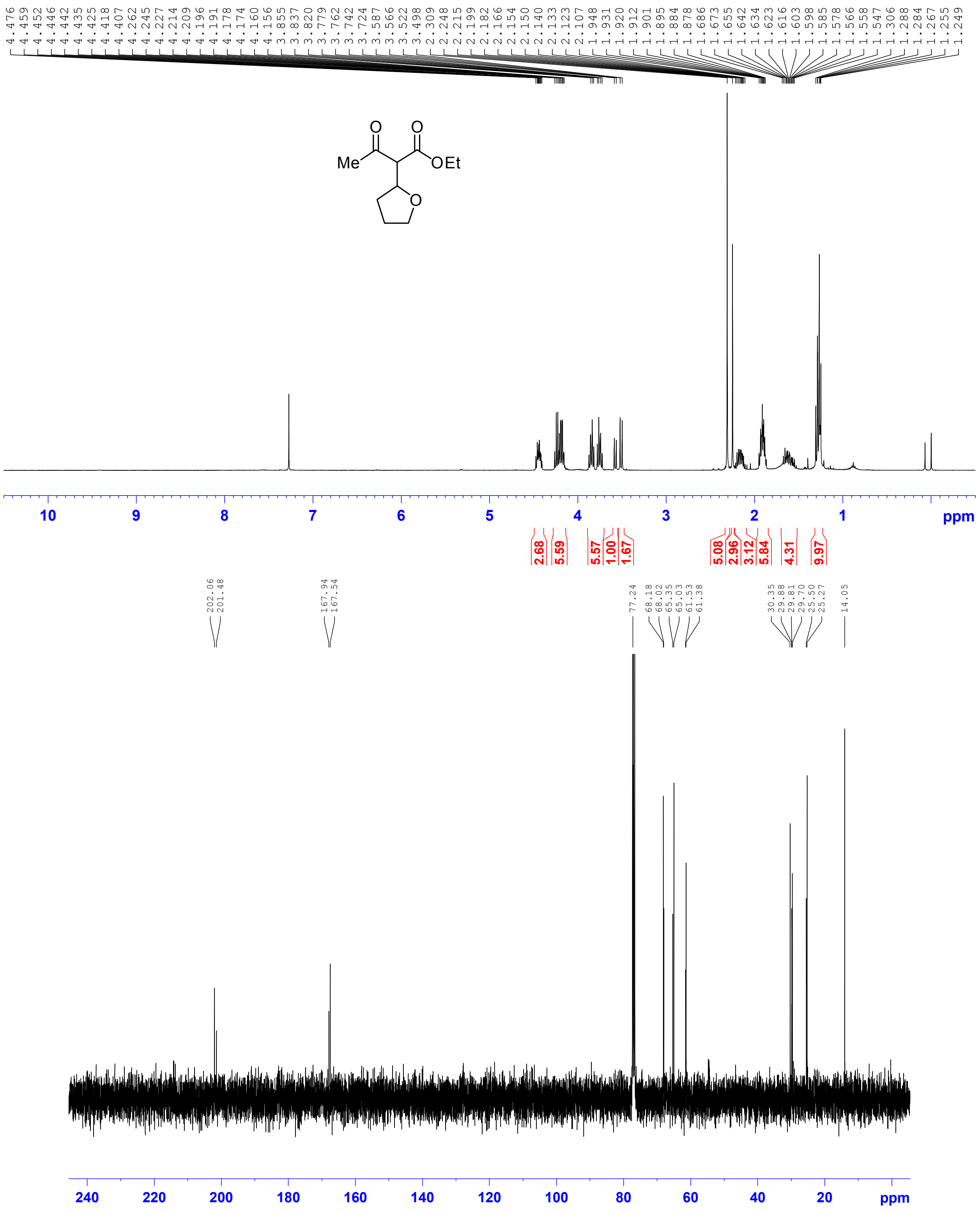

Figure S9. ^{1}H and ^{13}C NMR spectra of ethyl 3-oxo-2-(tetrahydrofuran-2-yl)butanoate (**4i**).

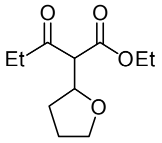

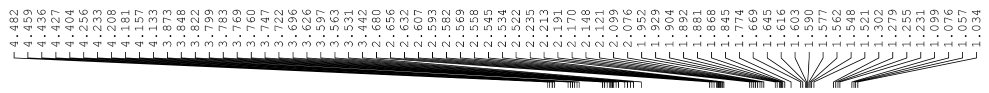

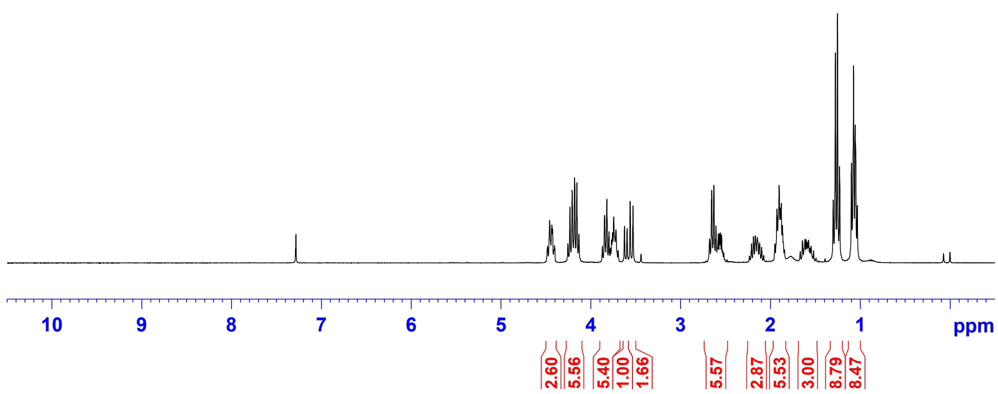

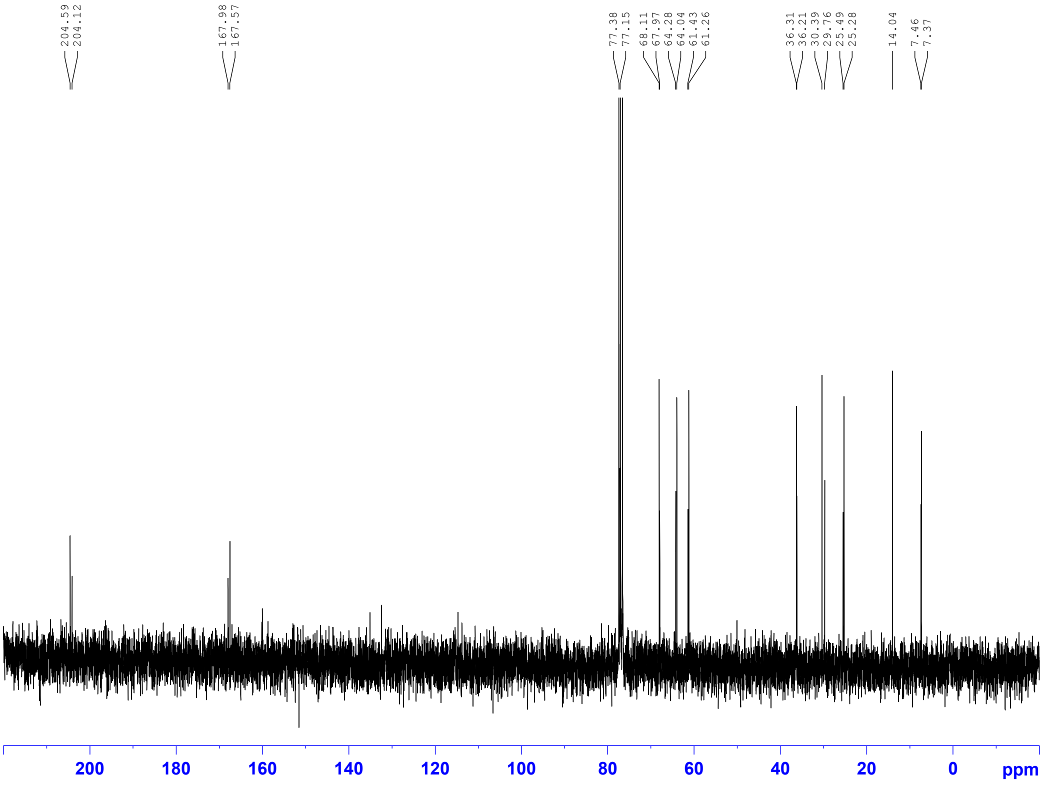

Figure S10. ^{1}H and ^{13}C NMR spectra of ethyl 3-oxo-2-(tetrahydrofuran-2-yl)pentanoate (**4j**).

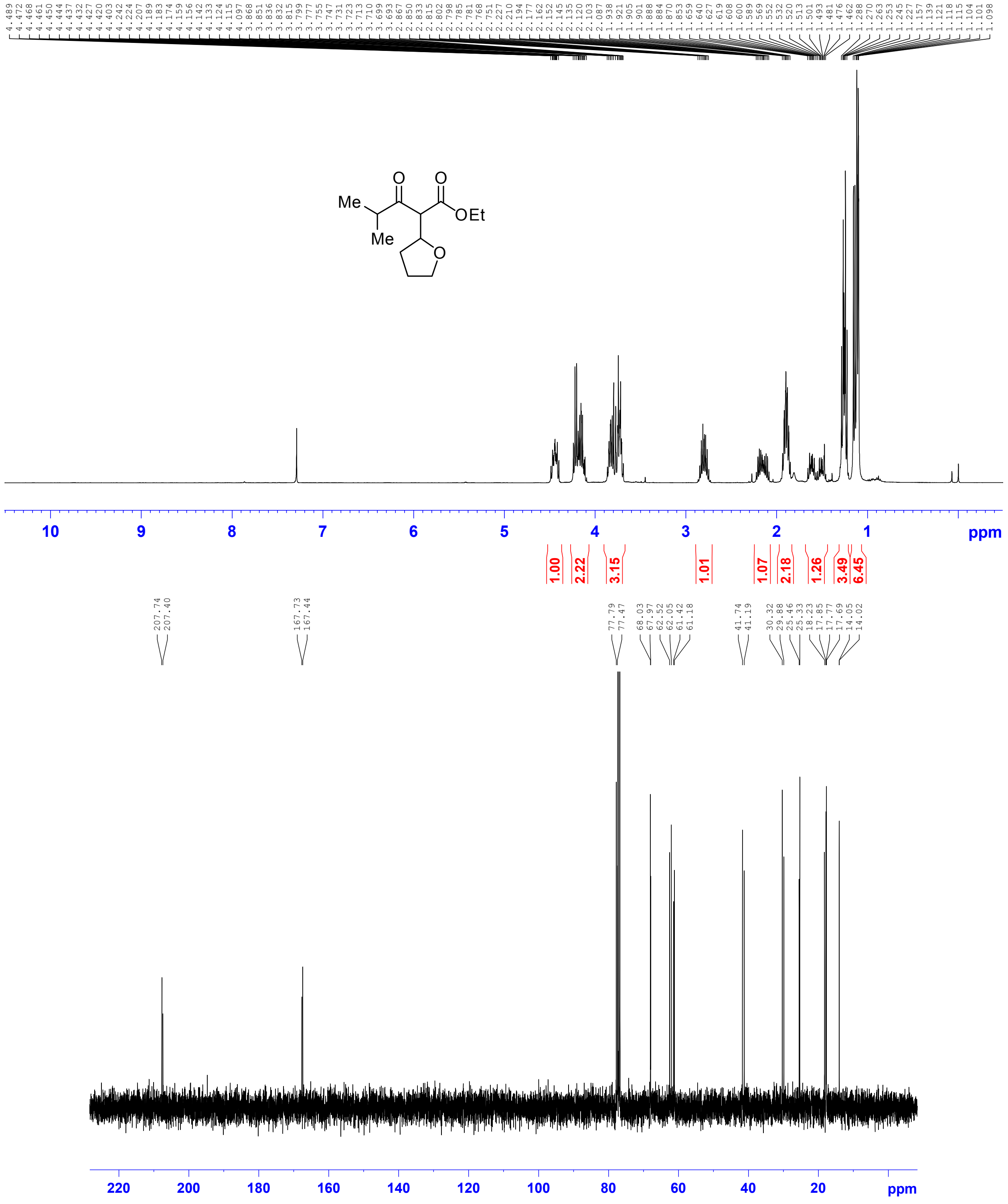

Figure S11. ^{1}H and ^{13}C NMR spectra of ethyl 4-methyl-3-oxo-2-(tetrahydrofuran-2-yl) pentanoate (**4k**).

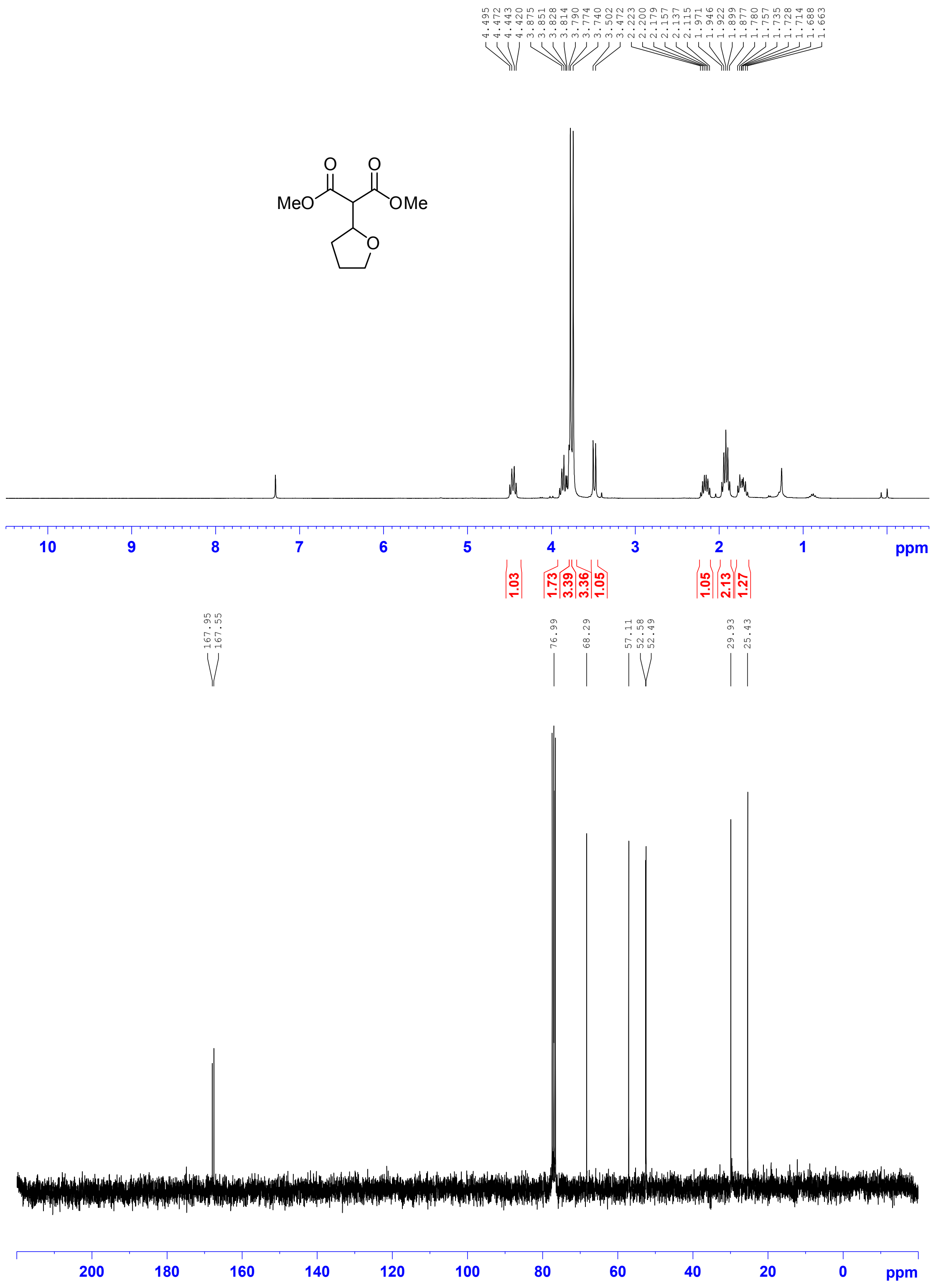

Figure S12. ^{1}H and ^{13}C NMR spectra of dimethyl 2-(tetrahydrofuran-2-yl)malonate (**4l**).

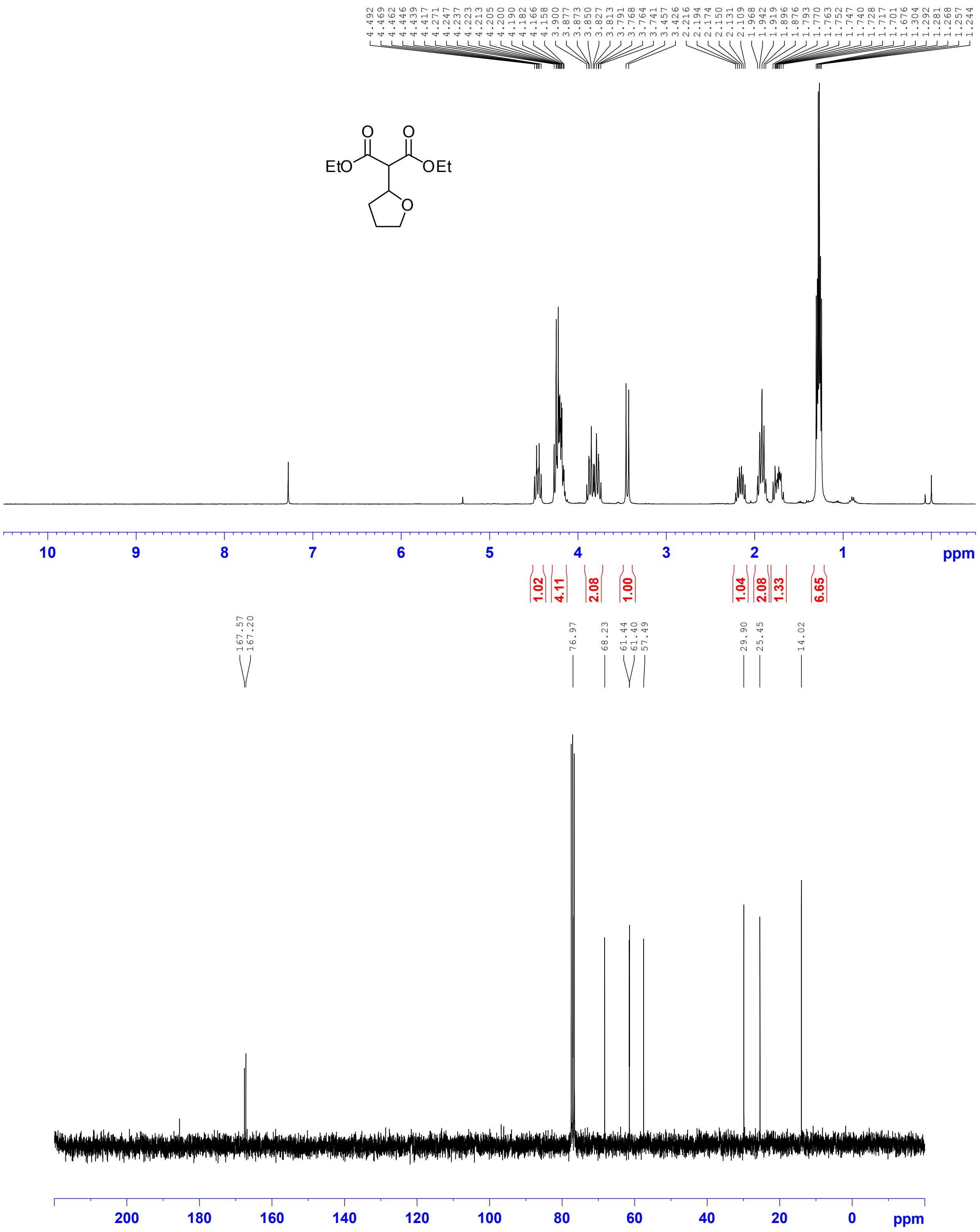

Figure S13. ^{1}H and ^{13}C NMR spectra of diethyl 2-(tetrahydrofuran-2-yl)malonate (**4m**).

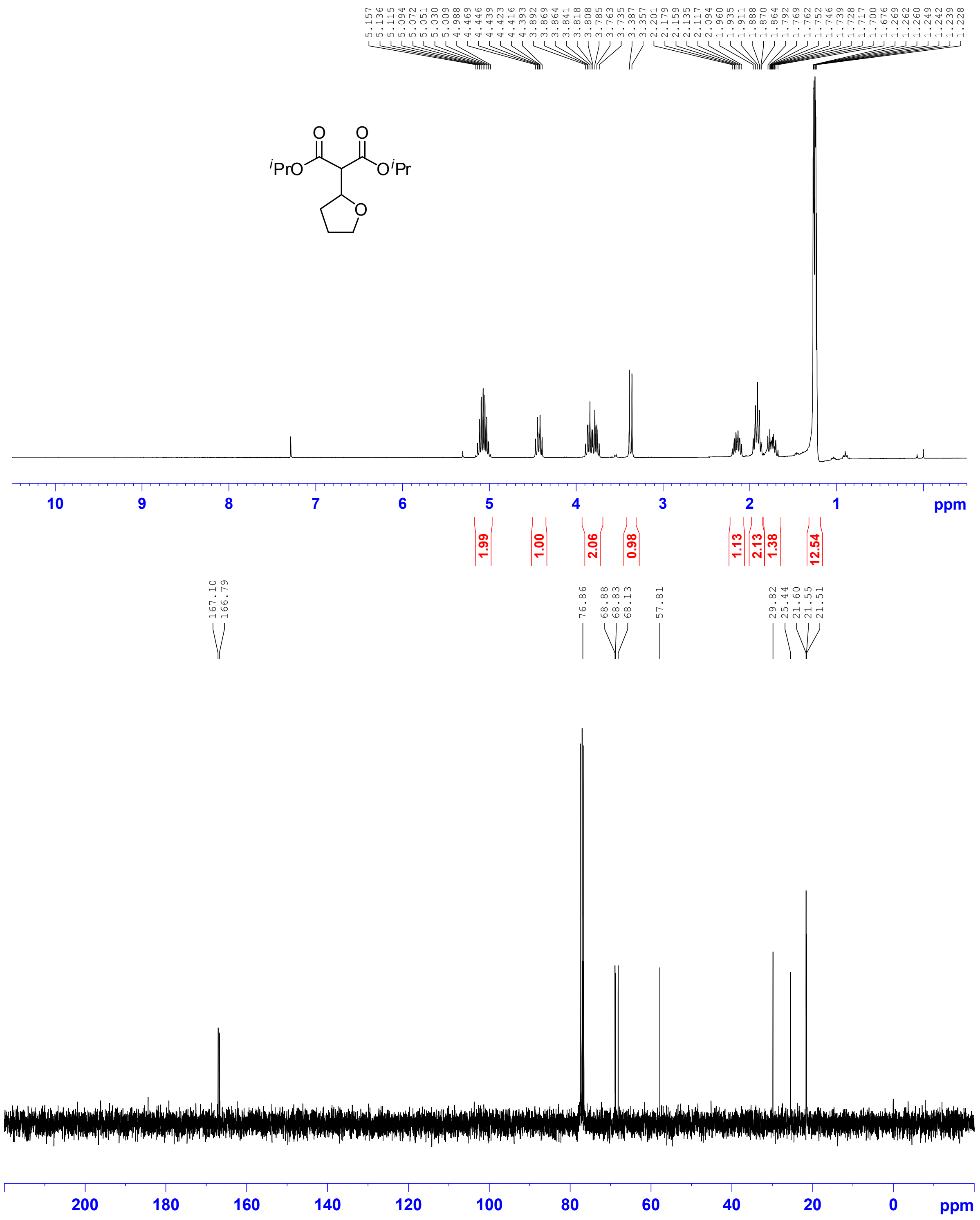

Figure S14. ^{1}H and ^{13}C NMR spectra of diisopropyl 2-(tetrahydrofuran-2-yl)malonate (**4n**).

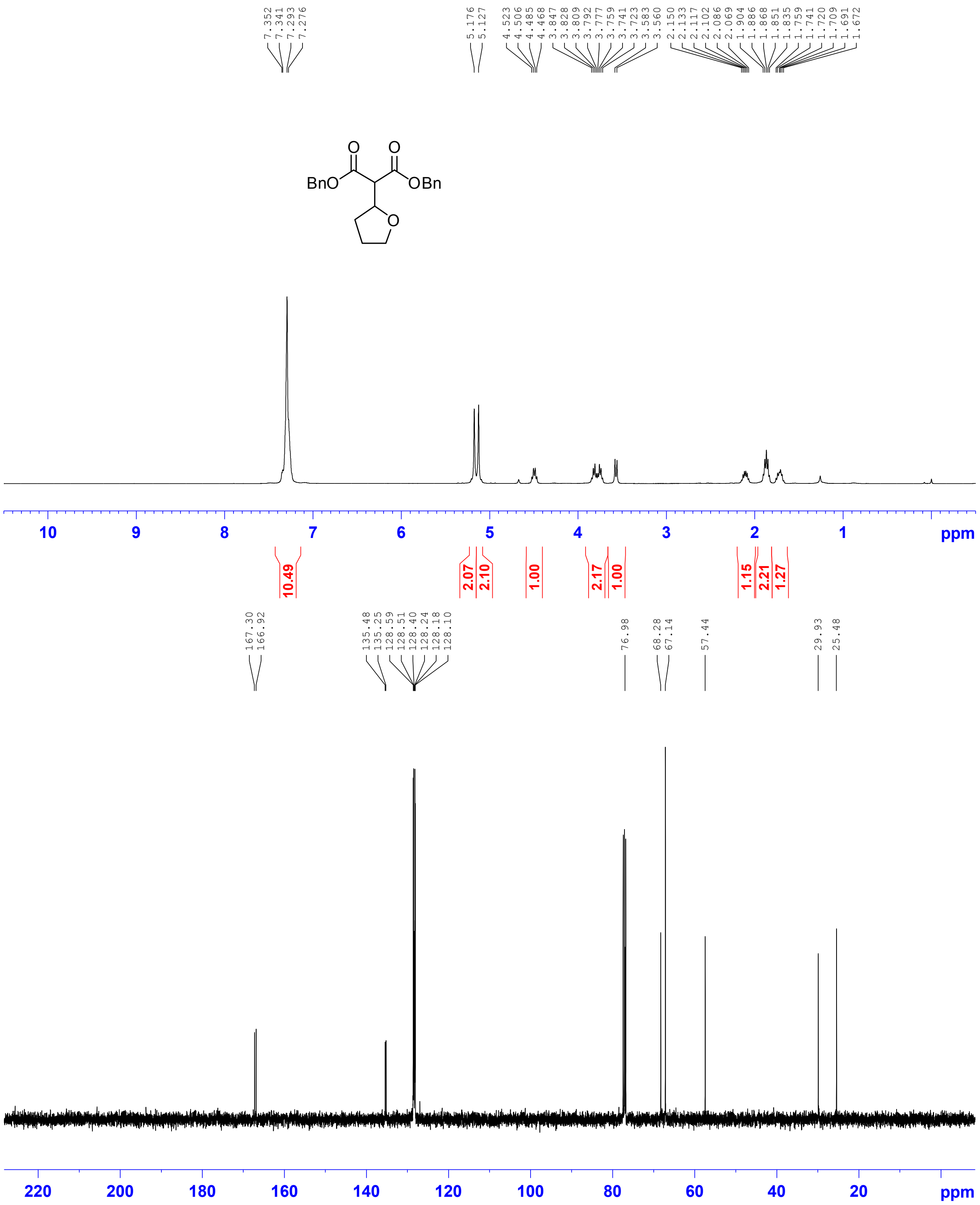

Figure S15. ^{1}H and ^{13}C NMR spectra of dibenzyl 2-(tetrahydrofuran-2-yl)malonate (**4o**).

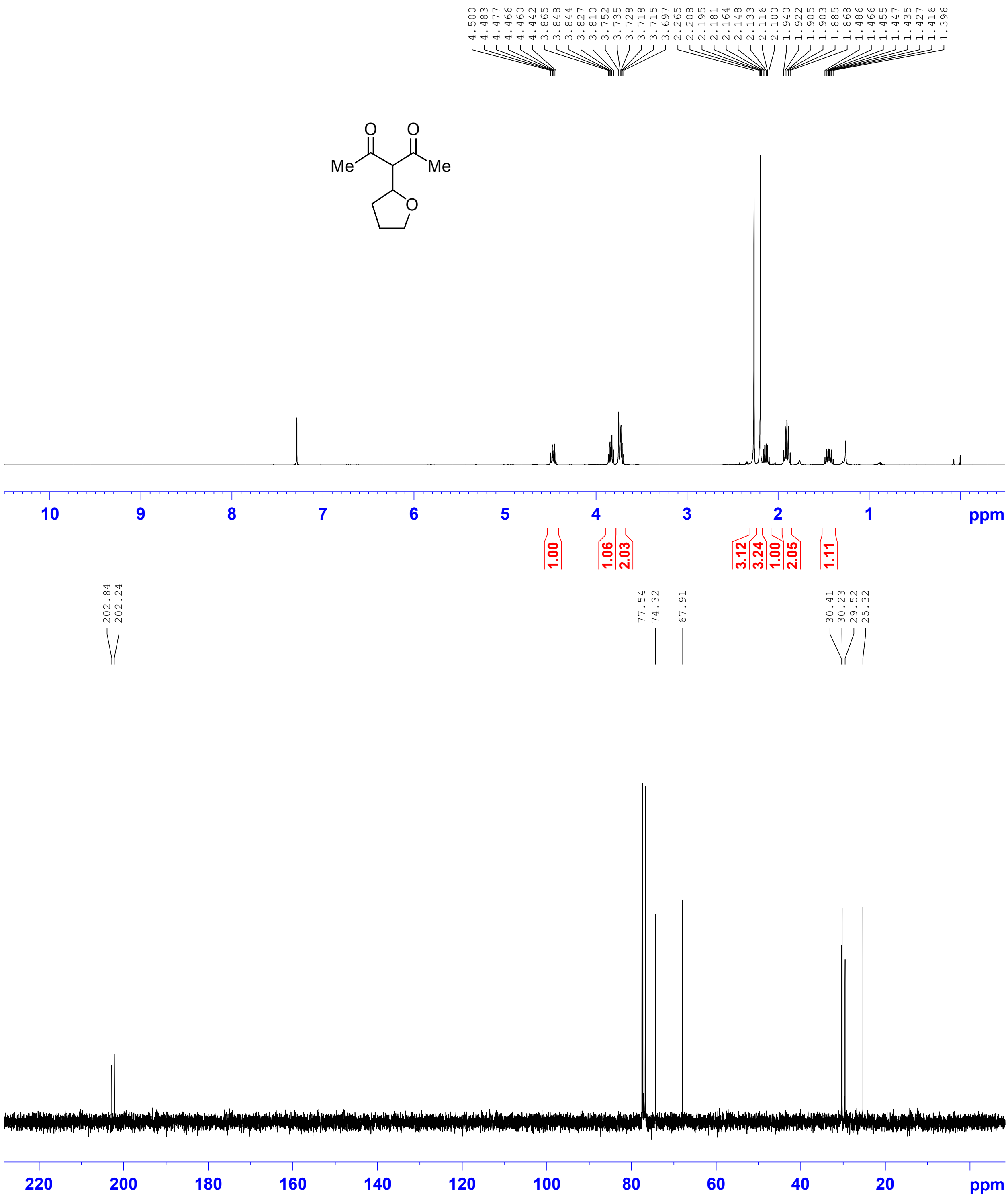

Figure S16. ^{1}H and ^{13}C NMR spectra of 3-(tetrahydrofuran-2-yl)pentane-2,4-dione (**4p**).

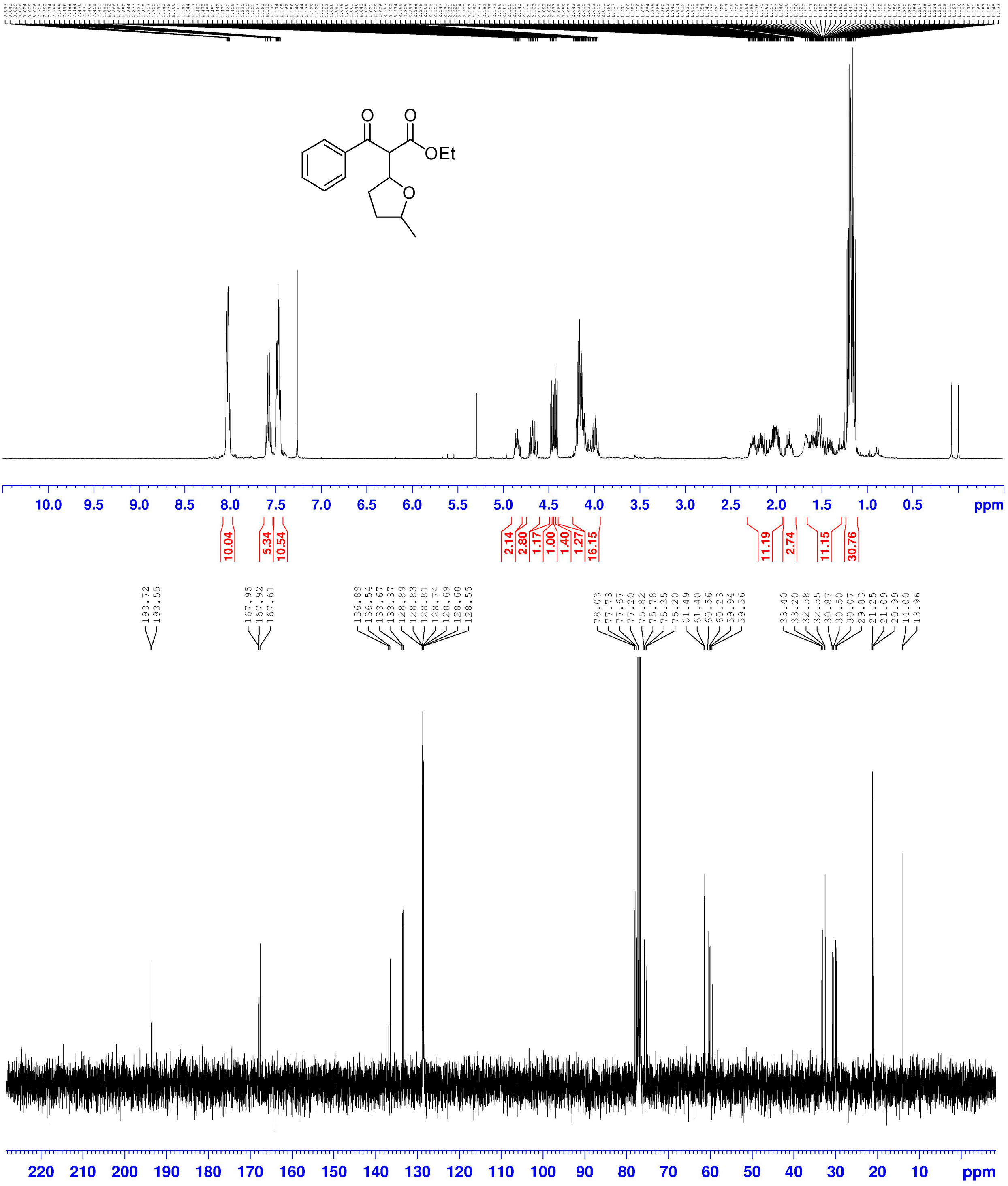

Figure S17. ^{1}H and ^{13}C NMR spectra of ethyl 2-(5-methyltetrahydrofuran-2-yl)-3-oxo-3-phenylpropanoate (**4q**).

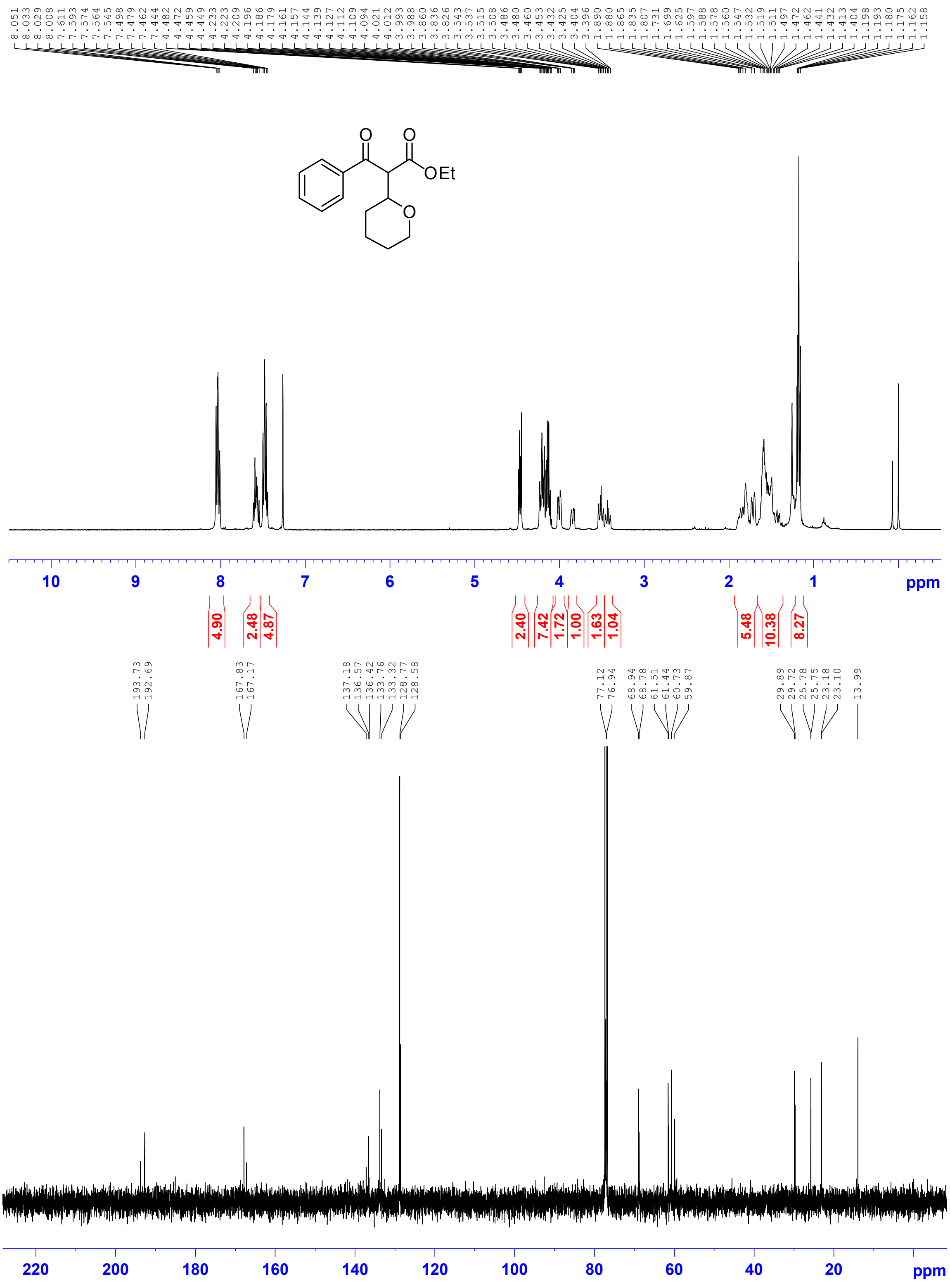

Figure S18. ^{1}H and ^{13}C NMR spectra of ethyl 3-oxo-3-phenyl-2-(tetrahydro-2H-pyran-2-yl) propanoate (**4r**).

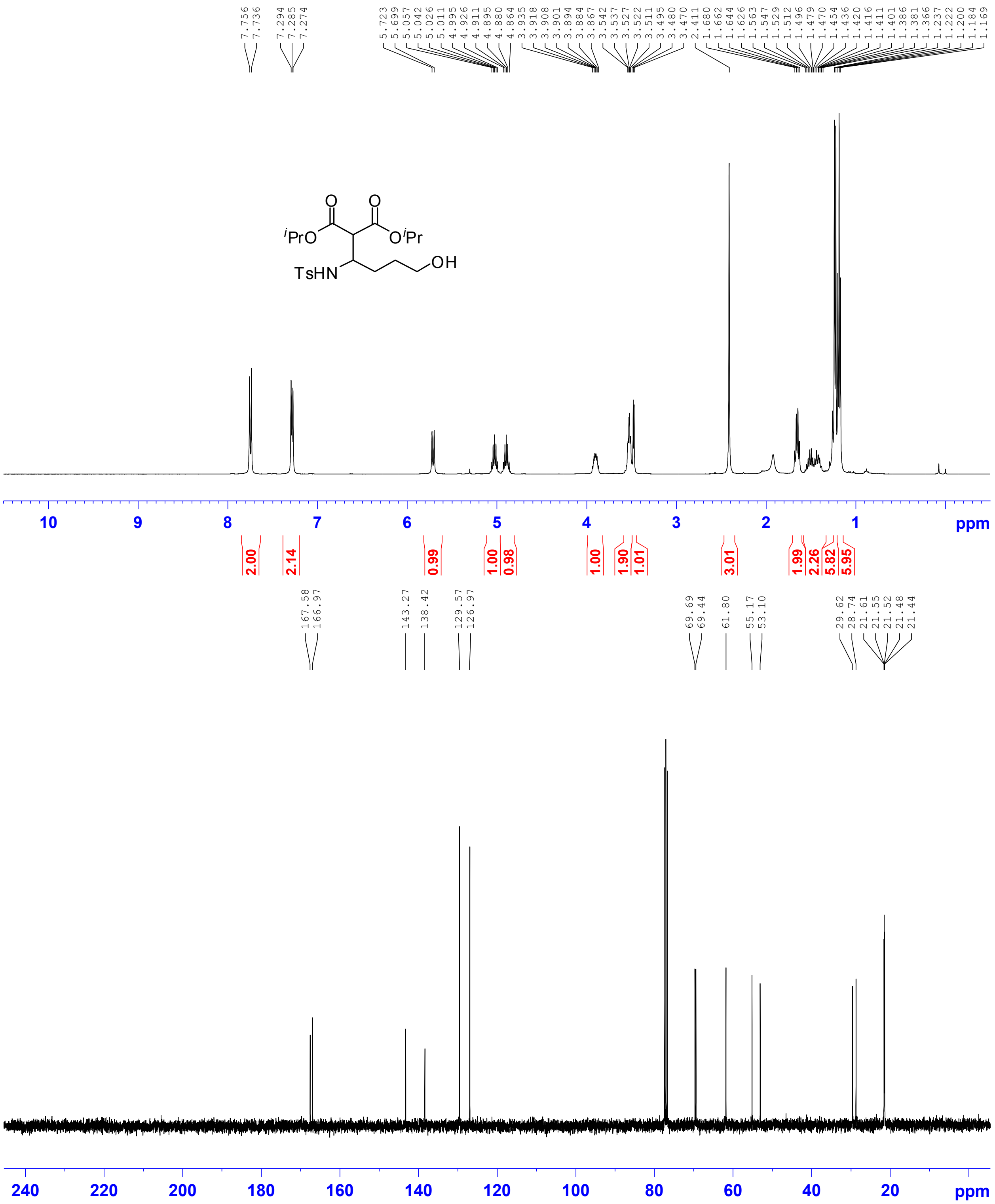

Figure S19. ^{1}H and ^{13}C NMR spectra of diisopropyl 2-(4-hydroxy-1-(4-methylphenyl sulfonamido)butyl)malonate (**5n**).
